# Computationally repurposing drugs for breast cancer subtypes using a network-based approach

**DOI:** 10.1186/s12859-022-04662-6

**Published:** 2022-04-20

**Authors:** Forough Firoozbakht, Iman Rezaeian, Luis Rueda, Alioune Ngom

**Affiliations:** 1 School of Computer Science, University of Windsor, 401 Sunset Ave., Windsor, ON Canada; 2 Rocket Innovation Studio, 156 Chatham St W, Windsor, ON Canada

**Keywords:** Drug repurposing, Network-based approach, Drug-disease network

## Abstract

*‘De novo’* drug discovery is costly, slow, and with high risk. Repurposing known drugs for treatment of other diseases offers a fast, low-cost/risk and highly-efficient method toward development of efficacious treatments. The emergence of large-scale heterogeneous biomolecular networks, molecular, chemical and bioactivity data, and genomic and phenotypic data of pharmacological compounds is enabling the development of new area of drug repurposing called ‘in silico’ drug repurposing, i.e., computational drug repurposing (CDR). The aim of CDR is to discover new indications for an existing drug (drug-centric) or to identify effective drugs for a disease (disease-centric). Both drug-centric and disease-centric approaches have the common challenge of either assessing the similarity or connections between drugs and diseases. However, traditional CDR is fraught with many challenges due to the underlying complex pharmacology and biology of diseases, genes, and drugs, as well as the complexity of their associations. As such, capturing highly non-linear associations among drugs, genes, diseases by most existing CDR methods has been challenging. We propose a network-based integration approach that can best capture knowledge (and complex relationships) contained within and between drugs, genes and disease data. A network-based machine learning approach is applied thereafter by using the extracted knowledge and relationships in order to identify single and pair of approved or experimental drugs with potential therapeutic effects on different breast cancer subtypes. Indeed, further clinical analysis is needed to confirm the therapeutic effects of identified drugs on each breast cancer subtype.

## Introduction

Discovery of a new drug, especially for cancer can be a very time-consuming and costly process. It normally takes between 10 to 15 years to develop a new drug [1] and can cost upward of tens of billion dollars [2–4]. On the other hand, the success rate of developing a new drug for cancer is very low [5], and the number of new FDA-approved drugs has been declining since past 25 years [6].

Drug repositioning and repurposing are effective alternative strategies to find new uses of existing drugs. Both drug repositioning and repurposing processes consist of using an existing drug for treatment of a disease other than its primary or initial purpose. If the drug is already FDA-approved, the process is called drug repurposing, while if the drug is in trial or experimental phase, the process is called drug repositioning. Since in this work we use the same methodology for all existing drugs, irrespective of their FDA-approval status, for simplicity, we refer to the methodology as “drug repurposing” in a general sense.

In case of repurposed drugs (and to a lesser degree for repositioned drugs, depending on their trial or experimental stage), the overall cost and time associated with using it for treatment of other diseases is significantly lower than developing a new drug [4].

In order to repurpose an existing drug for a new disease, the main challenge is to identify new relationships between drugs and diseases. To overcome this challenge, a variety of approaches have been introduced including computational, biological and experimental approaches, as well as hybrid schemes that combine both computational and biological techniques. Computational approaches for drug repurposing bear much lower cost and other barriers in comparison to biological experimental approaches, which makes it a more appealing strategy and a very good starting point for further clinical trials and biological validations [7].

The majority of existing computational methods for drug repurposing are based on the comparison between gene expression response of various cell lines before and after treatment or a combination of several types of data corresponding to various aspects of disease-drug relationships [8–11]. For example, Lotfi et al. grouped drug repurposing methods based on their principle source of biological data and core methodology, including gene regulatory networks, metabolic networks and molecular interaction networks [9], while Zou et al. categorized drug repurposing methods into two groups of data-driven and hypothesis-driven approaches [11]. Xue et al., on the other hand, focused on the underlying methodology used in drug repurposing, when it regards to categorizing those methods [12]. Luo et al. used Singular Value Thresholding (SVT) to predict scores for unknown drug-disease pairs based on known relationship between drugs and diseases [13]. Zhang et al. utilized a drug similarity network, a disease similarity network, and known drug-disease associations to explore the potential associations among unrelated pairs of drugs and diseases [14].

Generally speaking, we can group drug repurposing approaches into three distinct groups: text-mining approaches [15–21], semantics-based approaches [22–24], and finally network-based approaches [25–36]. The latter takes into the account the relationship and interactions between genes in their corresponding pathways. For example Bourdakou et al. used statistical co-expression networks to highlight and prioritize genes for breast cancer subtypes and leveraging them for drug repurposing [36]. One of the biggest difference between the proposed framework and the previous network-based methods is the ability of the proposed framework to identify not only single drugs, but also pairs of combined drugs (and theoretically unlimited number of drug combinations) for a given disease with a reasonable computational overhead, which enables it to find combinations of drugs that could far outreach the therapeutic effects of single drugs for a given breast cancer subtype (or any other disease in general).

This paper introduces a novel network-based approach to identify drugs with the highest repurposability with respect to each of ten breast cancer subtypes. This goal is achieved by first finding driver genes responsible for each subtype using genomic and transcriptomic data, which are then used along with pathway data in order to find those drugs that have the highest repurposing scores for each of ten breast cancer subtypes. The results show that the proposed method is able to identify potential effective known and experimental drugs developed for other diseases to be repurposed for various breast cancer subtypes. Indeed, further wet lab analysis is needed to determine the therapeutic level of identified drugs on each breast cancer subtype. For reference, what we refer to here as ten breast cancer subtypes are ten distinctive sub-groups identified in [37].

Moreover, we used the proposed method to identify single and pairs of drugs for Triple Negative (TN) breast cancer tumors. Between 10% to 15% of breast cancer cases are considered as TN, where they lack any hormone epidermal growth factor receptor 2 (HER-2), estrogen receptors (ER), and progesterone receptors (PR) in the tumor [38]. Thus, the traditional targeted (often hormone) therapy that targets one of these hormones are ineffective in these cases. This lack of targeted therapies has intensified the interest in this group of patients. Our results show that the proposed method were able to computationally identify single and paired repurposed drugs that could have therapeutic effect on this this group.

## Materials and methods

For drug expression data, we used level-5 data of the LINCS dataset (from Gene Expression Omnibus with the reference number GSE70138), which consists of *z*-score values of more than 118,000 drug/ concentration/ treatment_time for more than 12,000 genes. In order to make the process more computationally manageable, we used only the lowest and highest dosages of each drug (generally 0.04 and 10.0 μmol, correspondingly) and a default 24-h time-point frame for the analysis, in case of having more than one time-point frame. If a drug does not have a 24-h time-point frame, we use the default time-point frame indicated in LINCS database.

### Obtaining candidate genes

**Fig. 1:**
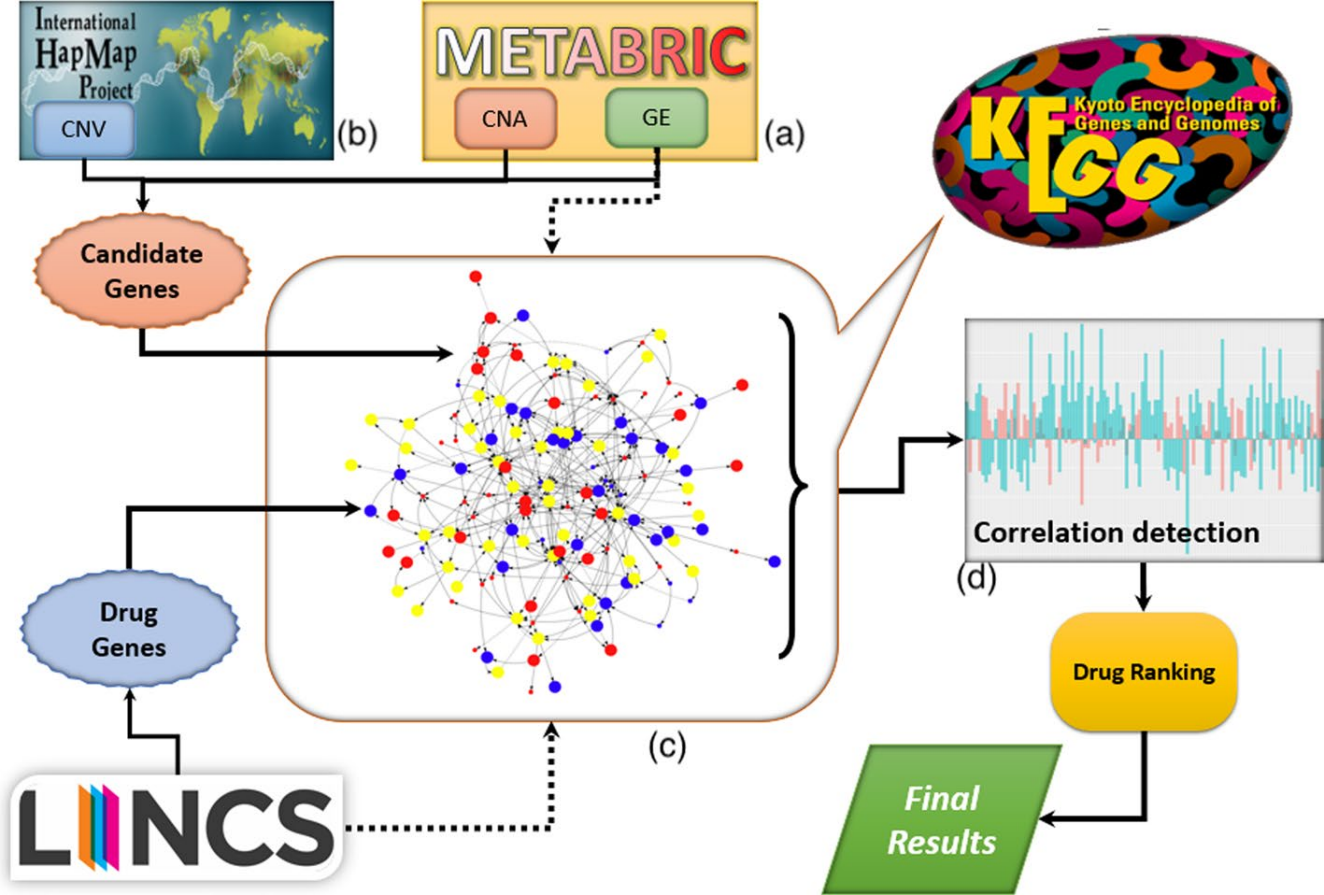
Schematic view of the proposed framework for identification of best repurposing drugs for each breast cancer subtype. METABRIC dataset is used to obtain copy number aberration and gene expression data for breast cancer subtypes. HapMap data is used to obtain copy number variation information. Lincs dataset is used to obtain the effect of different drug compounds on gene expression of cancer samples. KEGG dataset is used to create a universal pathway network [44]

In the first step, we use CNA, CNV and GE data to find the most informative genes, separately for each subtype. To do so, we first use CNA/CNV information to find those genes that have very high genotypic aberration in each subtype based on their GISTIC score [39]. GISTIC identifies significant aberrations using two steps. In the first step, it calculates the G-score statistic, which involves both the frequency of occurrence and the amplitude of the aberration. In the second step, it assesses the significance of each aberration using Fisher’s Exact test [40]. these two steps take place in Fig. 1a. To make sure that we only target aberrations in the copy number and not common variations across different populations, we use the HapMap database [41] (shown in Fig. 1b. HapMap is a catalog of common genetic variants that occur in human. We only consider those genes for a significant test that have CNA but no CNV. We also use gene expression data to identify the top differentially expressed genes for each subtype. For this, we used Chi2 [42] to rank genes based on their ability to separate each subtype from the remaining subtypes. At the end, after obtaining the top genes using CNA/CNV and GE data separately, if CNA/CNV analysis determined N genes as significant in terms of their genomic aberrations, we select the top N genes from GE data; then out of these two gene sets, we take the intersection as candidate genes. These candidate genes are those genes that have both significant differences in terms of gene expression and copy number aberrations.

### Obtaining gene scores

The measurement is a normalized *z*-score value for each replicate of a given gene treated with the same perturbation agent (i.e., perturbagen: either drugs or small molecule compounds or others) based on 95% confidence level [43]. Thus, for each pair of gene and drug agent, we consider a value between − 10 and 10. A value close to zero shows that the expression of a given gene will not be affected by the drug agent. In comparison, based on the concepts of gene expression inhibition and induction, a negative or positive score shows that the expression of the given gene decreases or increases, respectively, because of the effect of the drug agent.

We have used the METABRIC dataset [37], which contains the copy number values and gene expression levels of 2000 primary breast tumors with long-term clinical follow-up. It can be accessed from the European Genome-Phenome Archive using the accession number EGAS00000000083. In [37], the copy number aberrations and copy number variations generated using Affymetrix SNP 6.0 arrays and gene expression data were obtained using Illumina HT 12 technology. The dataset contains two sets of data, *validation* set and *discovery* set. Due to the lack of class labels in the validation set, in this paper we only use the discovery set, which contains 997 samples from ten subtypes of breast cancer. Each sample contains expression data for 48,803 probe IDs. The expression of all probes corresponding to the same gene have been merged based on the median expression of those probes, which maps all the probes to 24,351 unigenes. We calculate the same normalized *z*-score values for each of the ten breast cancer subtypes in the METABRIC dataset, (which can be accessed from European Genome-Phenome Archive with the study id EGAS00000000083), in such a way that the normalized *z*-score of each gene is a value between -10 and 10. A value close to zero shows that the expression of a given gene will not be affected by the disease, while a negative or positive score shows that the expression of the given gene decreases or increases, respectively, because of the effect of the disease.

### Creating unified global human pathway

In the next step, we use the KEGG Pathway database to find all possible paths between genes [44]. A biological pathway is a series of actions among molecules in a cell that leads to a certain product or a change in the cell. It can trigger the assembly of new molecules, such as a fat or protein, turn genes on and off, or spur a cell to move [45]. The version of the KEGG Pathway database we used contains 265 human pathways. So, by taking union of all genes and also all existing direct relations between each pair of genes, we create a unified global human pathway (UGHP). The UGHP contains interaction between 4985 genes in all 265 human pathways in KEGG as a matrix, where $UGHP_{ij}$ represents signaling interaction type between gene *i* and gene *j*. The values of the matrix could be -1, 1 or 0 representing activation, suppression, or no direct signal from gene *i* to gene *j*.

### Calculating drug-disease repurposing score

At this point, for each drug $D_i$ and breast cancer subtype $S_j$, we perform the following steps:

1. Select the top 50 affected genes by the drug $D_i$ from the LINCS dataset by ranking the genes based on their absolute *z*-score values and call them *drug genes*. Note that at the end of the process, the pipeline focuses only on negative correlations between drug and disease.
2. Use the candidate genes corresponding to subtype $S_j$ that we identified in using copy number alteration (CNA), copy number variation (CNV) and gene expression (GE) data [46]. We call these candidate genes *disease genes*.
3. Map back these drug and disease genes to UGHP to create a drug-disease network, $D_i S_j$, which contains the shortest paths between each pair of drug-disease genes (shown in Fig. 1c). Thus, the maximum number of nodes in $D_i S_j$ network, *N*, is given as follows:
  1 $$\begin{aligned} N = G_{dr}+G_{di}+G_i \end{aligned}$$
  where $G_{dr}$ is the number of drug genes, $G_{di}$ is the number of disease genes, and $G_i$ is the number of intermediate genes in the shortest path between each pair of drug and disease genes.
4. Since for each gene in this drug-disease network we have two *z*-score values (one for the effect of drug and one for the effect of disease), we construct two arrays, one consists of drug *z*-score values while the other consists of disease *z*-score values with identical gene order.
5. We compute Pearson correlation [47, 48] between the above arrays using the following formula (shown in Fig. 1d):
  2 $$\begin{aligned} r = \frac{ \sum _{i=1}^{n}(x_i-{\bar{x}})(y_i-{\bar{y}}) }{ \sqrt{\sum _{i=1}^{n}(x_i-{\bar{x}})^2}\sqrt{\sum _{i=1}^{n}(y_i-{\bar{y}})^2}} \end{aligned}$$
  where $x_i$ and ${\overline{x}}$ are the *z*-score value of gene *i* and average of all *z*-score values for the drug group in drug-disease network, and $y_i$ and ${\overline{y}}$ are the *z*-score value of gene *i* and average of all *z*-score values for the disease group in the drug-disease network, respectively.

Figure 1 depicts the proposed framework for the identification of best repurposing drugs for each breast cancer subtype.

Obtaining a positive correlation between a given drug genes and the disease genes means that the drug and the disease have similar effect on the genes in the drug-disease network. In contrast, obtaining a negative correlation implies that the drug’s effect on the genes in the drug-disease network is opposite to the effect of the disease on the genes in that network.

Obtaining a negative correlation is a favorable case in this context, because we are looking for drugs that could have a potential reverse effect on the genes affected by the disease.

### Identifying combinations of drugs for repurposing

In the previous section, we solely focused on effects of each individual perturbation agent on each subtype of breast cancer. In this section, we test the hypothesis that combination of two or more drugs might be more effective in terms of reversing the effect of the disease, i.e., by generating a more negative correlation with the disease than each drug independently. For simplicity, in this step, we assume that the *z*-score value of a given pair of drugs to be additive with respect to the *z*-score value of each of those drugs independently. In other words, if we assume that the *z*-score value of drug $D_i$ on gene *G* is $X_i$, and the *z*-score value of drug $D_j$ on the same gene *G* is $X_j$, we can then assume that the *z*-score value for the given pair of drugs [$D_i$,$D_j$] is *X*, where $X=X_i + X_j$.

**Fig. 2:**
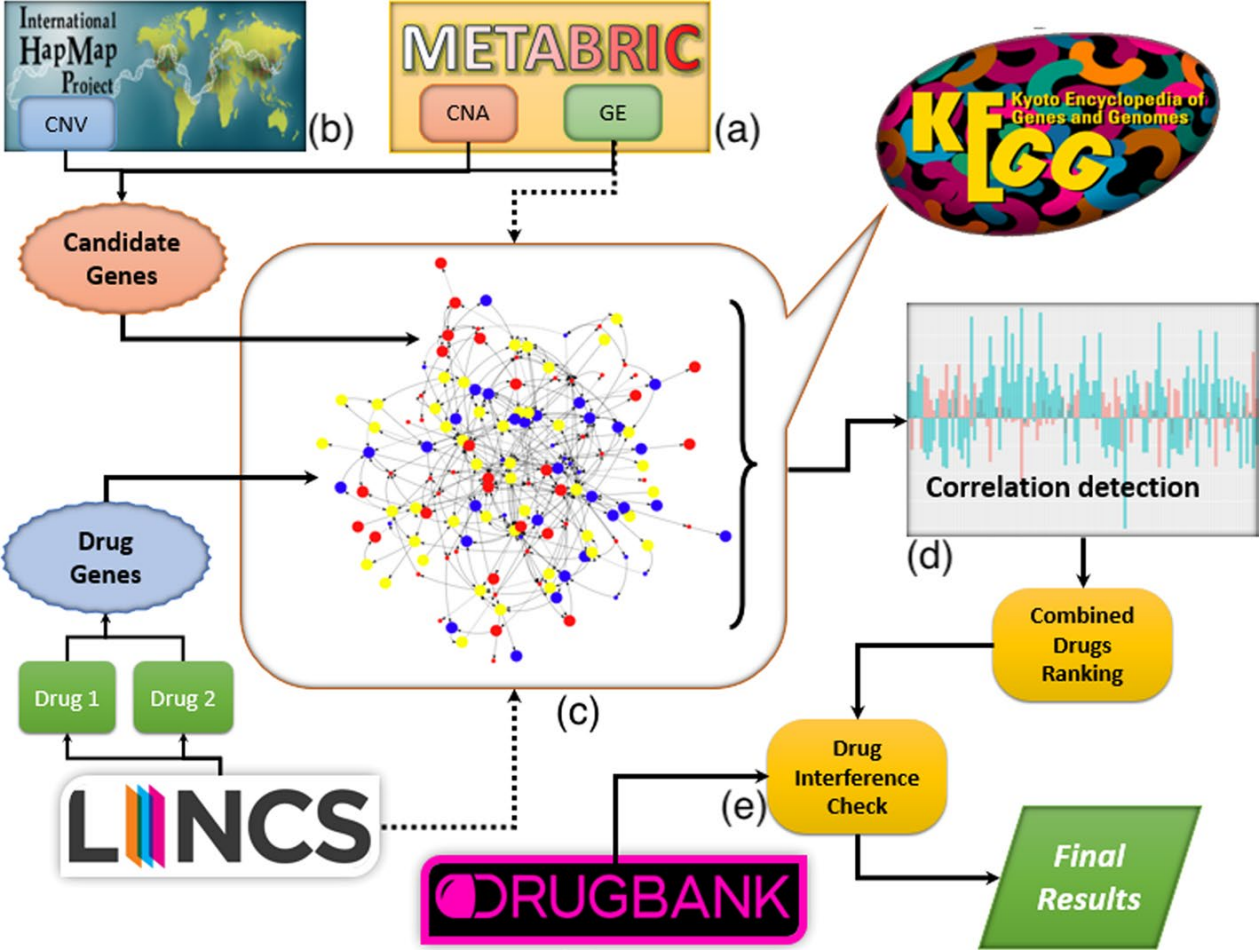
Schematic view of the proposed framework for identification of best pair of repurposing drugs for each breast cancer subtype. METABRIC dataset is used to obtain copy number aberration and gene expression data for breast cancer subtypes. HapMap data is used to obtain copy number variation information. Lincs dataset is used to obtain the effect of different drug compounds on gene expression of cancer samples. KEGG dataset is used to create a universal pathway network [44]. And finally, DrugBank’s drug interference checker is used to check any possible interference between each pair of drugs

Thus, in order to find the best repurposed pair of drugs for a given subtype of breast cancer, first, we calculate the combined *z*-score value of all genes for every pair of drugs, and then we pick the top genes with the highest absolute value of their combined *z*-score. Figure 2 depicts the proposed framework for identification of the best pair of repurposed drugs for each breast cancer subtype.

### Calculating drug-disease repurposing score for a pair of drugs

For pair of drug $D_i$ and $D_j$ and breast cancer subtype $S_k$, we perform the following steps:

1. Calculate the combined *z*-score value of all genes for pair of drugs $D_{ij}$, and selecting the top 50 genes with the highest absolute value of their combined *z*-score values as *paired drug genes*.
2. Use the candidate genes corresponding to subtype $S_k$ that we identified in using copy number alteration (CNA), copy number variation (CNV) and gene expression (GE) data [46]. We call these candidate genes *disease genes*.
3. Map back these drug and disease genes to UGHP to create a drug-disease network, $D_{ij} S_k$, which contains the shortest paths between each pair of drug-disease genes (shown in Fig. 2c). Thus, the maximum number of nodes in $D_{ij} S_k$ network, *N*, is given as follows:
  3 $$\begin{aligned} N = G_{dr}+G_{di}+G_{ij} \end{aligned}$$
  where $G_{dr}$ is the number of drug genes, $G_{di}$ is the number of disease genes, and $G_{ij}$ is the number of intermediate genes in the shortest path between each pair of combined drug and disease gene.
4. Since for each gene in this drug-disease network we have two *z*-score values (one for the effect of drug and one for the effect of disease), we construct two arrays, one consists of drug *z*-score values while the other consists of disease *z*-score values with identical gene order.
5. We compute Pearson correlation [47, 48] between the above arrays using the following formula (shown in Fig. 2d):
  4 $$\begin{aligned} r = \frac{ \sum _{i=1}^{n}(x_i-{\bar{x}})(y_i-{\bar{y}}) }{ \sqrt{\sum _{i=1}^{n}(x_i-{\bar{x}})^2}\sqrt{\sum _{i=1}^{n}(y_i-{\bar{y}})^2}} \end{aligned}$$
  where $x_i$ and ${\overline{x}}$ are the *z*-score value of gene *i* and average of all *z*-score values for the drug group in drug-disease network, and $y_i$ and ${\overline{y}}$ are the *z*-score value of gene *i* and average of all *z*-score values for the disease group in the drug-disease network, respectively.
6. Finally, we perform a post-verification analysis on drug interference for the identified pairs of drugs using *DrugBank’s Interaction Checker* tool, in order to confirm if there is any known interference between any of those identified pairs. (shown in Fig. 2e).
  A drug-drug interference is a situation in which one drug affects the activity of another. Drugs may interact with each other to cause side effects that are unexpected or unintended. If any pair of drugs have known drug-drug interference, we remove them from the analysis. For example, the combination of Tadalafil and palbociclib generated a negative correlation of -0.65 with subtype 3, which put them in the top 10 list of paired-drugs for this subtype. But given the fact that they have a known moderate interaction with each other, this pair has been removed from the analysis [49]. The reason for doing a post-verification analysis instead of checking it as a pre-process step, is that the post-verification approach gives us the flexibility of updating the results with newly discovered drug interference in the future without a need to rerun the analysis. Also, using a post-verification approach gives us the ability to deal with interferences between a given pair of drugs at different levels depending on the type or level of interference.

### Extension to triple-negative breast cancer tumors

In this section, we leverage the proposed pipeline for the ten breast cancer subtypes considered in the previous section, to identify potential repurposable drugs specifically for triple negative beast cancer tumors. In order to do so for identifying candidate genes, we treat triple negative samples in METABRIC as one group and all the remaining samples as another group. By running the pipeline introduced in [46], we identify the most discriminative genes in terms of gene expression and copy number aberration between TN and non-TN groups. Then, using the pipelines depicted in Figs.  1 and 2, we identify the top repurposing single and paired drugs for the TNBC subtype.

## Results and discussion

For reference, Table 1 shows the list of drugs that have been approved by FDA to date for breast cancer treatment [50]. The results show that the proposed model is able to identify highly negative correlated drugs corresponding to each of ten breast cancer subtypes, both when used in single drug mode or for identifying pairs of drugs. Some of the well-known and widely used breast cancer drugs have been identified among the top drugs, which again shows that the proposed approach was able to pick up current drugs with high accuracy. For example, Goserelin (Zoladex) is a well known and FDA approved hormone therapy drug for treatment of BC that showed up in top ten drugs for subtypes 2 and 8. Also, Palbociclib (Ibrance) is another well known and FDA approved chemo therapy drug for treatment of BC that showed up in top ten drugs for subtype 4. Moreover, Ruxolitinib, which showed up in top ten drugs for 9 out of 10 subtypes (Table 12) has been under several trials and studies regarding its potential inhibiting effects on BC [51, 52].

**Table 1:** List of FDA-approved drugs for breast cancer treatment

Abemaciclib	Kadcyla (Ado-Trastuzumab Emtansine)
Abitrexate (Methotrexate)	Kisqali (Ribociclib)
Abraxane (Paclitaxel Albumin-stabilized Nanoparticle Formulation)	Lapatinib Ditosylate
Ado-Trastuzumab Emtansine	Letrozole
Afinitor (Everolimus)	Lynparza (Olaparib)
Anastrozole	Megestrol Acetate
Aredia (Pamidronate Disodium)	Methotrexate
Arimidex (Anastrozole)	Methotrexate LPF (Methotrexate)
Aromasin (Exemestane)	Mexate (Methotrexate)
Capecitabine	Mexate-AQ (Methotrexate)
Clafen (Cyclophosphamide)	Neosar (Cyclophosphamide)
Cyclophosphamide	Neratinib Maleate
Cytoxan (Cyclophosphamide)	Nerlynx (Neratinib Maleate)
Docetaxel	Nolvadex (Tamoxifen Citrate)
Doxorubicin Hydrochloride	Olaparib
Ellence (Epirubicin Hydrochloride)	Paclitaxel
Epirubicin Hydrochloride	Paclitaxel Albumin-stabilized Nanoparticle Formulation
Eribulin Mesylate	Palbociclib
Everolimus	Pamidronate Disodium
Exemestane	Perjeta (Pertuzumab)
5-FU (Fluorouracil Injection)	Pertuzumab
Fareston (Toremifene)	Ribociclib
Faslodex (Fulvestrant)	Tamoxifen Citrate
Femara (Letrozole)	Taxol (Paclitaxel)
Fluorouracil Injection	Taxotere (Docetaxel)
Folex (Methotrexate)	Thiotepa
Folex PFS (Methotrexate)	Toremifene
Fulvestrant	Trastuzumab
Gemcitabine Hydrochloride	Tykerb (Lapatinib Ditosylate)
Gemzar (Gemcitabine Hydrochloride)	Velban (Vinblastine Sulfate)
Goserelin Acetate	Velsar (Vinblastine Sulfate)
Halaven (Eribulin Mesylate)	Verzenio (Abemaciclib)
Herceptin (Trastuzumab)	Vinblastine Sulfate
Ibrance (Palbociclib)	Xeloda (Capecitabine)
Ixabepilone	Zoladex (Goserelin Acetate)
Ixempra (Ixabepilone)	

### Single drug repurposing

**Fig. 3:**
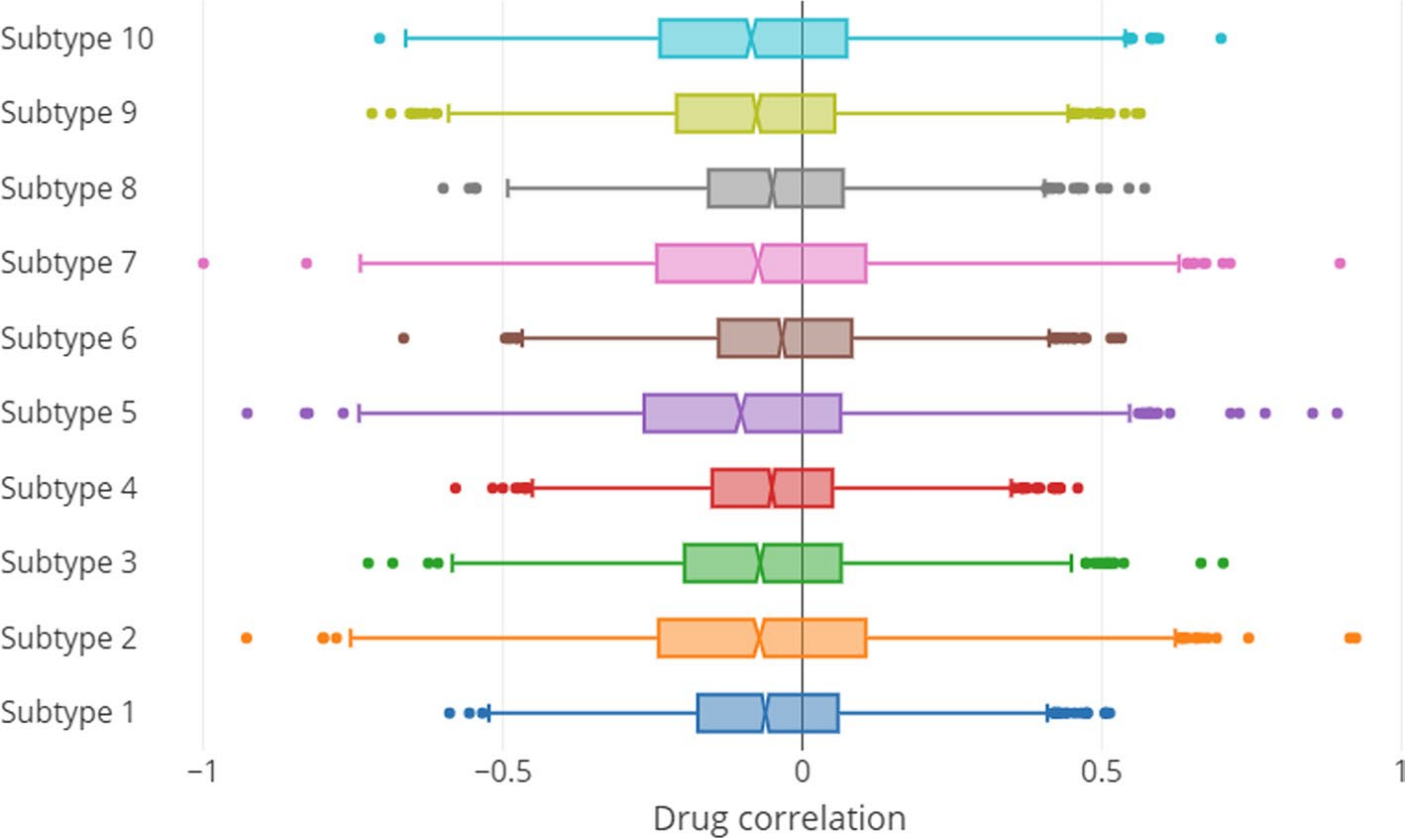
Distribution of drug-disease correlation for 3742 drugs across 10 breast cancer subtypes

Figure 3 shows the distribution of drug repurposing scores across the ten breast cancer subtypes. There are a few interesting observations. First, the response level of different BC subtypes to tested drugs are different. While the distribution of correlation scores among the tested drugs versus some of the subtypes such as subtypes 1, 4 and 6 are relatively narrow (which implies relatively lower response level of the aforementioned subtypes to the tested drugs), in some other subtypes, such as subtypes 2 and 8, we observe a wider distribution of these scores. This shows that effects of tested drugs could be widely different across subtypes. The second observation is regarding the median of these scores. As shown in the figure, in all subtypes, we observe a slight distribution bias toward negative repurposing scores, which implies that the tested drugs tend to exhibit more of a therapeutic effect than adverse effect.

Tables 2, 3, 4, 5, 6, 7, 8, 9, 10 and 11 show the top 20 inhibiting drugs corresponding to each of the ten subtypes. These drugs fall into three categories. *Experimental* drugs are those that are at the pre-clinical or at an animal testing stage. *Investigational* drugs are those that are in stage I, II or III of human clinical trials. Finally, *Approved* drugs are those drugs that have already been approved by FDA to be used for treatment of various diseases. Drugs that are FDA approved to be used for BC treatment (i.e. those listed in Table 1) have been highlighted in bold. Also, reference column lists any publication that suggested usage of that drug for BC treatment.

**Table 2:** Top 20 drugs corresponding to subtype 1

Rank	Drug	Dosage (μm)	Treatment time (h)	Score	Drug type	References
1	Ruxolitinib	10	24	− 0.589	Approved	[51, 52]
2	Ribavirin	10	24	− 0.556	Approved	[65]
3	Deferiprone	0.04	24	− 0.534	Approved	[66, 67]
4	Tranilast	0.04	24	− 0.523	Investigational	[68]
5	Tadalafil	10	24	− 0.521	Approved	
6	Rimexolone	0.04	24	− 0.515	Approved	
7	Bardoxolone methyl	0.04	24	− 0.511	Investigational	[69, 70]
8	Sirolimus	0.04	24	− 0.507	Approved	[71, 72]
9	Crizotinib	0.04	24	− 0.503	Approved	[73, 74]
10	PF-04217903	0.04	24	− 0.499	Investigational	
11	Dofequidar	10	24	− 0.498	Experimental	[75, 76]
12	GSK-2636771	10	24	− 0.495	Investigational	
13	Edetic acid	10	24	− 0.494	Approved	
14	MK-1775	10	24	− 0.490	Investigational	[77]
15	MF-101	10	24	− 0.478	Investigational	
16	Ranolazine	0.04	24	− 0.476	Approved	[78]
17	Semaxanib	0.04	24	− 0.475	Experimental	[79]
18	Iniparib	10	24	− 0.474	Investigational	[80]
19	AKT-inhibitor 1/2	10	24	− 0.473	Experimental	
20	Rupatadine	0.04	24	− 0.473	Approved

**Table 3:** Top 20 drugs corresponding to subtype 2

Rank	Drug	Dosage (μm)	Treatment time (h)	Score	Drug type	References
1	Bromocriptine	0.04	24	− 0.93	Approved	[63]
2	**Goserelin-Acetate**	0.04	24	− 0.8	Approved	[81]
3	Ruxolitinib	10	24	− 0.8	Approved	[51, 52]
4	ICI-185,282	0.04	24	− 0.78	Experimental	
5	SDZ-NKT-343	10	24	− 0.75	Experimental	
6	Fingolimod	0.04	24	− 0.72	Approved	[82]
7	L-690330	10	24	− 0.71	Investigational	
8	PHA-767491	10	24	− 0.68	Experimental	[83]
9	Tolvaptan	10	24	− 0.67	Approved	
10	Ribavirin	10	24	− 0.65	Approved	[65]
11	Hymecromone	0.04	24	− 0.64	Investigational	[84]
12	Tranilast	0.04	24	− 0.64	Investigational	[68]
13	Sapitinib	10	24	− 0.64	Investigational	[85]
14	Deferiprone	0.04	24	− 0.63	Approved	[66, 67]
15	Mibampator	0.04	24	− 0.63	Investigational	
16	Citrulline	0.04	24	− 0.63	Investigational	
17	Lidocaine	0.04	24	− 0.63	Approved	[86, 87]
18	PD-173074	10	24	− 0.62	Experimental	[88]
19	Ibuprofen	10	24	− 0.62	Approved	[89, 90]
20	Garcinol	10	24	− 0.62	Experimental	[91, 92]

**Table 4:** Top 20 drugs corresponding to subtype 3

Rank	Drug	Dosage (μm)	Treatment time (h)	Score	Drug type	References
1	Bromocriptine	0.04	24	− 0.72	Approved	[63]
2	Ruxolitinib	10	24	− 0.68	Approved	[51, 52]
3	Deferiprone	0.04	24	− 0.62	Approved	[66, 67]
4	Tadalafil	10	24	− 0.61	Approved	
5	Sirolimus	0.04	24	− 0.58	Approved	[71, 72]
6	Rupatadine	0.04	24	− 0.58	Approved	
7	Ribavirin	10	24	− 0.57	Approved	[65]
8	Pha-767491	10	24	− 0.57	Experimental	[83]
9	Etofylline-Clofibrate	10	24	− 0.57	Approved	
10	EDTA	10	24	− 0.56	Approved	[93]
11	Tranilast	0.04	24	− 0.56	Investigational	[68]
12	MK-1775	10	24	− 0.56	Investigational	[77]
13	Raclopride	10	24	− 0.54	Investigational	
14	Iniparib	10	24	− 0.54	Investigational	[80]
15	Bexarotene	10	24	− 0.54	Approved	[94]
16	PF-04217903	0.04	24	− 0.54	Investigational	
17	Swainsonine	0.05	24	− 0.53	Experimental	[95]
18	JTC-801	0.04	24	− 0.53	Experimental	[96]
19	Ofloxacin	10	24	− 0.53	Approved	[97]
20	Mibampator	0.04	24	− 0.52	Investigational

**Table 5:** Top 20 drugs corresponding to subtype 4

Rank	Drug	Dosage (μm)	Treatment time (h)	Score	Drug type	References
1	Rupatadine	0.04	24	− 0.58	Approved	
2	Ribavirin	10	24	− 0.52	Approved	[65]
3	**Palbociclib**	0.04	24	− 0.5	Approved	[98]
4	Raclopride	10	24	− 0.48	Investigational	
5	PHA-793887	10	24	− 0.47	Investigational	
6	Bafilomycin A1	0.05	6	− 0.46	Experimental	
7	Raloxifene	0.04	24	− 0.46	Approved	[99]
8	EDTA	0.04	24	− 0.46	Approved	[93]
9	Maraviroc	10	24	− 0.46	Approved	[64]
10	Ebselen	10	24	− 0.45	Investigational	[100]
11	Dasatinib	10	24	− 0.45	Approved	[101]
12	Labetalol	0.04	24	− 0.45	Approved	
13	Amiprilose	0.04	24	− 0.44	Experimental	
14	Dofequidar	10	24	− 0.44	Investigational	[102]
15	Deferiprone	0.04	24	− 0.44	Approved	[66, 67]
16	Sapitinib	10	24	− 0.44	Investigational	[85]
17	Calcitriol	0.04	24	− 0.44	Approved	[103]
18	PD-153035	0.04	24	− 0.43	Investigational	[104]
19	Finasteride	10	24	− 0.43	Approved	[105]
20	Etofylline-Clofibrate	10	24	− 0.43	Approved

**Table 6:** Top 20 drugs corresponding to subtype 5

Rank	Drug	Dosage (μm)	Treatment time (h)	Score	Drug type	References
1	Ruxolitinib	10	24	− 0.93	Approved	[51, 52]
2	AMG-837	0.04	24	− 0.83	Experimental	
3	Citrulline	0.04	24	− 0.82	Investigational	
4	Loperamide	10	24	− 0.77	Approved	[106]
5	SDZ-NKT-343	10	24	− 0.74	Experimental	
6	TG-100801	0.04	24	− 0.72	Investigational	
7	Tranilast	0.04	24	− 0.72	Investigational	[68]
8	Bisoprolol	10	24	− 0.71	Approved	[107]
9	Ribavirin	10	24	− 0.7	Approved	[65]
10	PF-04217903	0.04	24	− 0.7	Investigational	
11	Sirolimus	0.04	24	− 0.69	Approved	[71, 72]
12	Lidocaine	0.04	24	− 0.69	Approved	[86, 87]
13	PHA-767491	10	24	− 0.68	Experimental	[83]
14	WZ-4-145	0.04	3	− 0.68	Experimental	
15	Etofylline-Clofibrate	10	24	− 0.67	Approved	
16	Fingolimod	0.04	24	− 0.67	Approved	[82]
17	ICI-185,282	0.04	24	− 0.67	Experimental	
18	EDTA	10	24	− 0.67	Approved	[93]
19	Labetalol	0.04	24	− 0.66	Approved	
20	MF-101	10	24	− 0.66	Experimental

**Table 7:** Top 20 drugs corresponding to subtype 6

Rank	Drug	Dosage (μm)	Treatment time (h)	Score	Drug type	References
1	Ruxolitinib	10	24	− 0.67	Approved	[51, 52]
2	Rupatadine	0.04	24	− 0.5	Approved	
3	Maraviroc	10	24	− 0.49	Approved	[64]
4	Deferiprone	0.04	24	− 0.49	Approved	[66, 67]
5	Phentermine	0.04	24	− 0.49	Approved	
6	Iniparib	10	24	− 0.48	Investigational	[108]
7	ICI-185,282	0.04	24	− 0.48	Experimental	
8	Racecadotril	0.04	24	− 0.48	Investigational	
9	Fingolimod	0.04	24	− 0.47	Approved	[82]
10	Amiprilose	0.04	24	− 0.47	Experimental	
11	Tranilast	0.04	24	− 0.46	Investigational	[68]
12	Mibampator	0.04	24	− 0.46	Investigational	
13	Favipiravir	0.04	24	− 0.45	Approved	
14	Selisistat	0.04	24	− 0.45	Experimental	
15	ZD-7288	10	24	− 0.45	Experimental	
16	Proglumide	10	24	− 0.44	Experimental	
17	TG-100801	0.04	24	− 0.44	Investigational	
18	Ranolazine	0.04	24	− 0.44	Approved	[78]
19	Semaxanib	0.04	24	− 0.44	Investigational	[79]
20	Ribavirin	10	24	− 0.44	Approved	[65]

**Table 8:** Top 20 drugs corresponding to subtype 7

Rank	Drug	Dosage (μm)	Treatment time (h)	Score	Drug type	References
1	Bromocriptine	0.04	24	− 1	Approved	[63]
2	Ruxolitinib	10	24	− 0.83	Approved	[51, 52]
3	Fingolimod	0.04	24	− 0.74	Approved	[82]
4	Tranilast	0.04	24	− 0.72	Investigational	[68]
5	Etofylline-Clofibrate	10	24	− 0.71	Approved	
6	SDZ-NKT-343	10	24	− 0.71	Experimental	
7	Isbufylline	0.04	24	− 0.71	Experimental	
8	Raloxifene	0.04	24	− 0.7	Approved	[99]
9	PHA-767491	10	24	− 0.69	Experimental	[83]
10	PF-04217903	0.04	24	− 0.69	Investigational	
11	TG-100801	0.04	24	− 0.69	Investigational	
12	L-690330	10	24	− 0.69	Investigational	
13	MK-1775	10	24	− 0.68	Investigational	[109, 110]
14	ICI-185,282	0.04	24	− 0.68	Experimental	
15	Rupatadine	0.04	24	− 0.67	Approved	
16	Hymecromone	0.04	24	− 0.66	Investigational	[84]
17	PD-173074	10	24	− 0.66	Experimental	[88]
18	MG-132	20	24	− 0.66	Experimental	[111]
19	Raclopride	10	24	− 0.65	Investigational	
20	Deferiprone	0.04	24	− 0.65	Approved	[66, 67]

**Table 9:** Top 20 drugs corresponding to subtype 8

Rank	Drug	Dosage (μm)	Treatment time (h)	Score	Drug type	References
1	Semaxanib	0.04	24	− 0.6	Investigational	[79]
2	Ruxolitinib	10	24	− 0.56	Approved	[51, 52]
3	**Goserelin-Acetate**	0.04	24	− 0.55	Approved	[81]
4	Raloxifene	0.04	24	− 0.54	Approved	[99]
5	XMD11-85h	0.04	3	− 0.49	Experimental	
6	Deferiprone	0.04	24	− 0.49	Approved	[66, 67]
7	Cinepazide	0.04	24	− 0.48	Investigational	
8	Ebselen	10	24	− 0.48	Investigational	[100]
9	WH-4-025	0.04	24	− 0.48	Experimental	
10	Nimesulide	0.04	24	− 0.47	Approved	[112]
11	Rupatadine	0.04	24	− 0.47	Approved	
12	Phentermine	0.04	24	− 0.47	Approved	
13	MF-101	10	24	− 0.47	Experimental	
14	Amiprilose	0.04	24	− 0.47	Experimental	
15	Etofylline-Clofibrate	10	24	− 0.47	Approved	
16	XMD-1150	10	3	− 0.46	Experimental	
17	Lidocaine	10	24	− 0.46	Approved	[86, 87]
18	TG-100801	0.04	24	− 0.46	Investigational	
19	Dasatinib	10	24	− 0.45	Approved	[101]
20	Apitolisib	10	24	− 0.45	Investigational	[113]

**Table 10:** Top 20 drugs corresponding to subtype 9

Rank	Drug	Dosage (μm)	Treatment time (h)	Score	Drug type	References
1	Rupatadine	0.04	24	− 0.72	Approved	
2	Ruxolitinib	10	24	− 0.69	Approved	[51, 52]
3	Raloxifene	0.04	24	− 0.65	Approved	[99]
4	Emtricitabine	10	24	− 0.65	Approved	[114]
5	L-690330	10	24	− 0.65	Investigational	
6	Tepotinib	10	24	− 0.64	Approved	[115]
7	Amiprilose	0.04	24	− 0.63	Experimental	
8	TG-100801	0.04	24	− 0.61	Investigational	
9	MG-132	20	24	− 0.61	Experimental	[111]
10	Belinostat	0.04	24	− 0.59	Approved	[116, 117]
11	Bromocriptine	0.04	24	− 0.59	Approved	[63]
12	Vidarabine	0.04	24	− 0.59	Approved	
13	Ranolazine	0.04	24	− 0.58	Approved	[78]
14	Lisinopril	0.04	24	− 0.58	Approved	[107]
15	PD-173074	10	24	− 0.57	Experimental	[88]
16	Vilazodone	10	24	− 0.57	Approved	[118]
17	Dexamethasone	0.04	24	− 0.57	Approved	[119, 120]
18	Semaxanib	0.04	24	− 0.57	Investigational	[79]
19	Mocetinostat	0.04	24	− 0.57	Investigational	[121]
20	Fingolimod	0.04	24	− 0.56	Approved	[82]

**Table 11:** Top 20 drugs corresponding to subtype 10

Rank	Drug	Dosage (μm)	Treatment time (h)	Score	Drug type	References
1	Ruxolitinib	10	24	− 0.71	Approved	[51, 52]
2	Bitopertin	10	24	− 0.66	Investigational	
3	ICI-185,282	0.04	24	− 0.66	Experimental	
4	Tranilast	0.04	24	− 0.65	Investigational	[68]
5	Bafilomycin A1	0.05	6	− 0.65	Experimental	
6	EDTA	10	24	− 0.64	Approved	[93]
7	MK-1775	10	24	− 0.63	Investigational	[109, 110]
8	Emtricitabine	10	24	− 0.63	Approved	[114]
9	Etofylline-Clofibrate	10	24	− 0.63	Approved	
10	PF-04217903	0.04	24	− 0.61	Investigational	
11	Sapitinib	10	24	− 0.61	Investigational	[85]
12	Bisoprolol	10	24	− 0.61	Approved	[107]
13	Fingolimod	0.04	24	− 0.61	Approved	[82]
14	XMD11-85h	0.04	3	− 0.61	Experimental	
15	PHA-767491	10	24	− 0.6	Experimental	[83]
16	Finasteride	10	24	− 0.6	Approved	[105]
17	Ribavirin	10	24	− 0.6	Approved	[65]
18	Labetalol	0.04	24	− 0.6	Approved	
19	MG-132	20	24	− 0.6	Experimental	[111]
20	Deferiprone	0.04	24	− 0.59	Approved	[66, 67]

Some of the drugs in these lists are well-known and have been used extensively for either breast cancer or other types of cancer. For example, *Raloxifene* is among the top ten drugs in most, if not all, of the ten subtypes. It was originally approved by FDA in 1997 for the management and prevention of osteoporosis in postmenopausal women and reduction in risk for invasive breast cancer. However, recent studies have shown that this drug might be effective for breast cancer treatments [53, 54]. Also, *Ruxolitinib*, which is among the top three drugs for all but subtype 4, was approved by the FDA for the treatment of patients with intermediate or high-risk myelofibrosis [55], though it is currently used in multiple clinical trials in patients with metastatic breast cancer as well [51, 56].

The findings discussed above show that the proposed method is able to correctly identify *Raloxifene* and *Ruxolitinib* drugs as very good candidates for most of the BC subtypes. We also observe investigational and experimental drugs in the list for each of the subtypes that could have therapeutic effects on each BC subtype. For example, *PHA-793887* is a potent inhibitor of multiple cyclin-dependent kinases such as CDK2, CDK5 and CDK7, and has been shown to possess the ability to affect the differentiation of melanoma cells. [57, 58]. This drug is currently in a clinical trial phase [59].

In another comparison, Table 12 shows the top 30 drugs ranked by their median score across all ten subtypes. As shown in the table, some drugs such as *Palbociclib* and *PHA-793887* demonstrate potential effectiveness across all of the subtypes by being ranked among the top drugs. In contrast, some others such as *Silmitasertib* and *Proglumide* demonstrate potential effectiveness in some of the subtypes, while being less effective in others.

**Table 12:** Rank comparison among the top 30 drugs across all 10 breast cancer subtypes

Overall Rank	Drugs	S1	S2	S3	S4	S5	S6	S7	S8	S9	S10	Median
1	Ruxolitinib	1	3	2	43	1	1	2	2	2	1	2
2	Tranilast	4	12	11	193	7	11	4	43	32	4	11
3	Rupatadine	22	44	6	1	31	2	15	11	1	51	13
4	Ribavirin	2	10	7	2	9	20	60	66	21	17	13.5
5	Deferiprone	3	14	3	15	27	4	20	6	183	20	14.5
6	Etofylline-Clofibrate	16	29	9	20	15	45	5	15	45	9	15.5
7	Fingolimod	36	6	58	137	16	9	3	109	20	13	18
8	ICI-185282	61	4	21	46	17	7	14	39	35	3	19
9	PF-04217903	10	25	16	398	10	40	10	107	25	10	20.5
10	Raloxifene	30	43	26	7	25	31	8	4	3	75	25.5
11	EDTA	13	55	10	8	18	127	112	196	38	6	28
12	Amiprilose	23	46	57	13	42	10	61	14	7	34	28.5
13	Bafilomycin A1	101	28	33	6	21	34	21	30	191	5	29
14	Dexamethasone	27	21	91	171	32	68	23	87	17	23	29.5
15	Dofequidar	11	88	24	14	106	29	104	29	31	56	30
16	MK-1775	14	49	12	61	24	37	13	46	41	7	30.5
17	TG-100801	121	50	44	60	6	17	11	18	8	53	31
18	Swainsonine	15	58	17	34	90	48	63	28	23	27	31
19	Raclopride	24	54	13	4	30	36	19	71	137	36	33
20	L-690330	37	7	38	35	61	32	12	314	5	133	36
21	Phentermine	57	22	43	57	135	5	36	12	26	55	39.5
22	PHA-767491	64	8	8	153	13	257	9	325	379	15	39.5
23	PD-173074	59	18	42	108	37	27	17	356	15	142	39.5
24	Lidocaine	122	17	103	212	12	88	25	17	51	30	40.5
25	Mibampator	98	15	20	22	93	12	46	60	85	37	41.5
26	PD-153035	26	108	214	18	35	128	33	47	235	39	43
27	AKT-inhibitor-1-2	21	24	40	110	48	57	34	150	282	22	44
28	Maraviroc	41	77	25	9	41	3	48	149	132	415	44.5
29	SDZ-NKT-343	104	5	65	203	5	39	6	51	241	24	45
30	Clomipramine	48	198	37	177	22	46	44	155	153	32	47

**Fig. 4:**
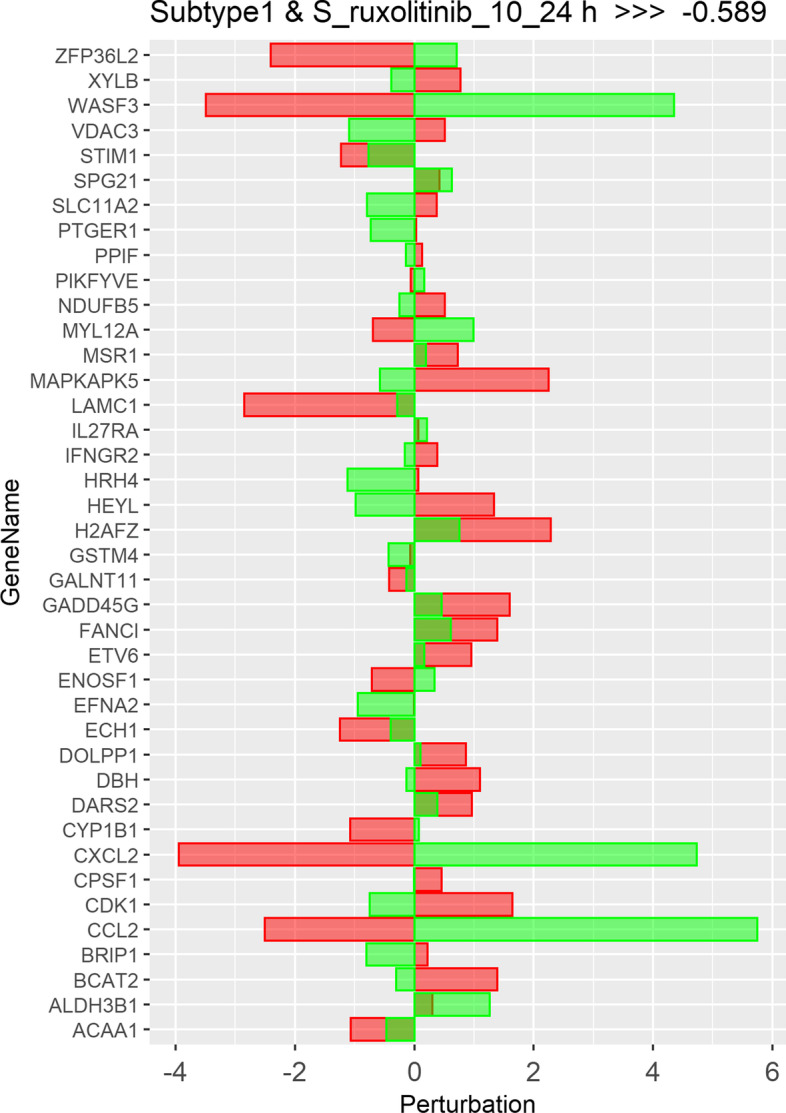
Perturbation scores across all genes involved in drug-disease network of top repurposed drug (Ruxolitinib) corresponding to subtype 1. Red bars depict the scores of subtype 1, while green bars depict the scores for the repurposed drug

**Fig. 5:**
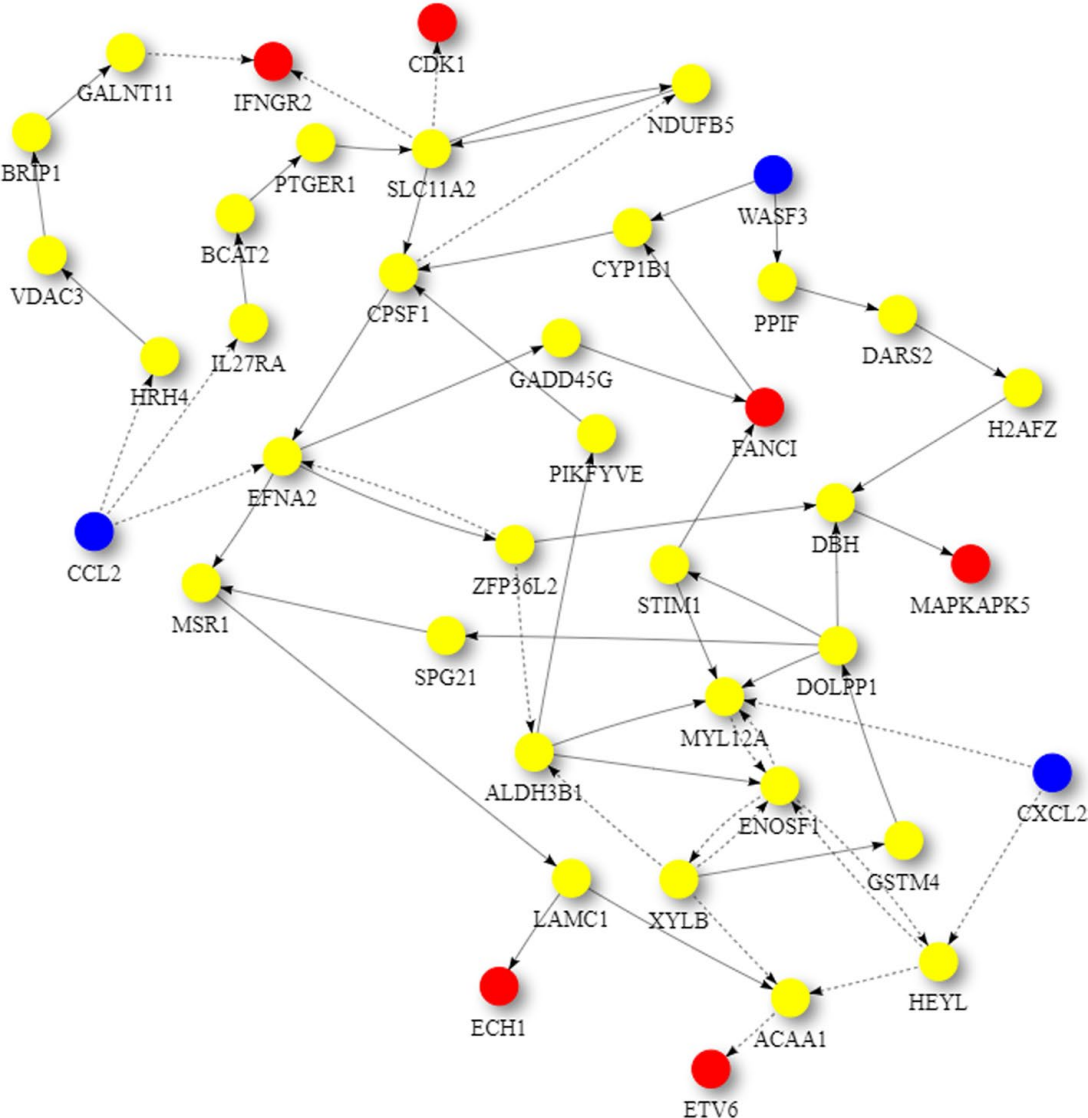
Unified Global Human Pathway (UGHP) subnetwork corresponding to the top repurposed drug (Ruxolitinib) and subtype 1. Blue nodes depict Drug related genes, while red nodes depict Subtype 1 related candidate genes involved in this drug-disease pathway

Also, Figs. 4 and 5 show the perturbation scores and drug-disease network of one of the top identified drugs, *Ruxolitinib*, for Subtype 1. *Ruxolitinib*, as mentioned earlier in this paper, is a small-molecule kinase inhibitor that is selective for the Janus Associated Kinases (JAK) 1 and 2, which are responsible for the mediation of cytokine and growth factor signaling, which, in turn, affects the immune function and hematopoiesis [60].

### Paired drug repurposing

**Fig. 6:**
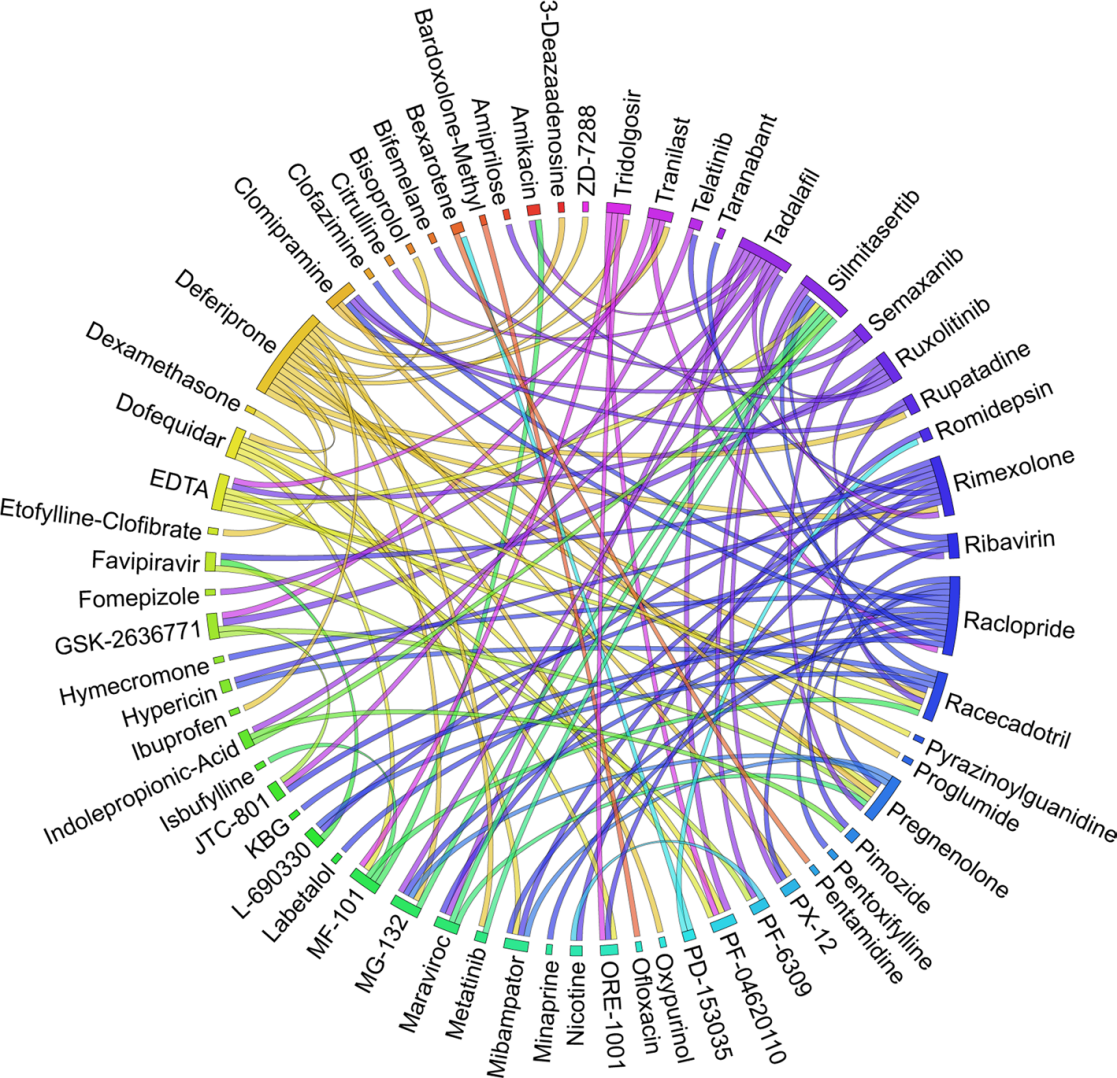
Top pairs of drugs with highest anti-correlation corresponding to subtype 1

**Fig. 7:**
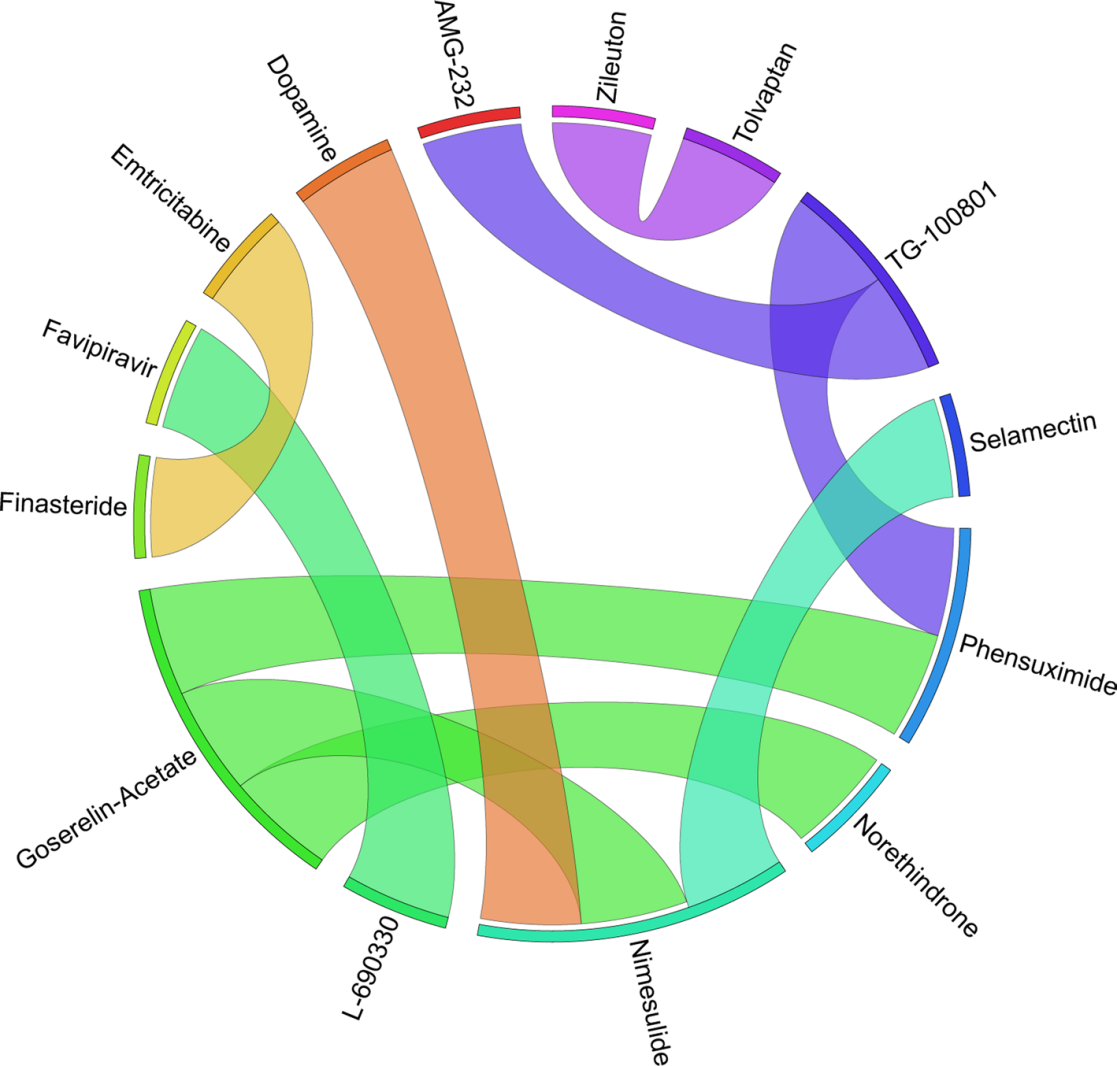
Top pairs of drugs with highest anti-correlation corresponding to subtype 2

**Fig. 8:**
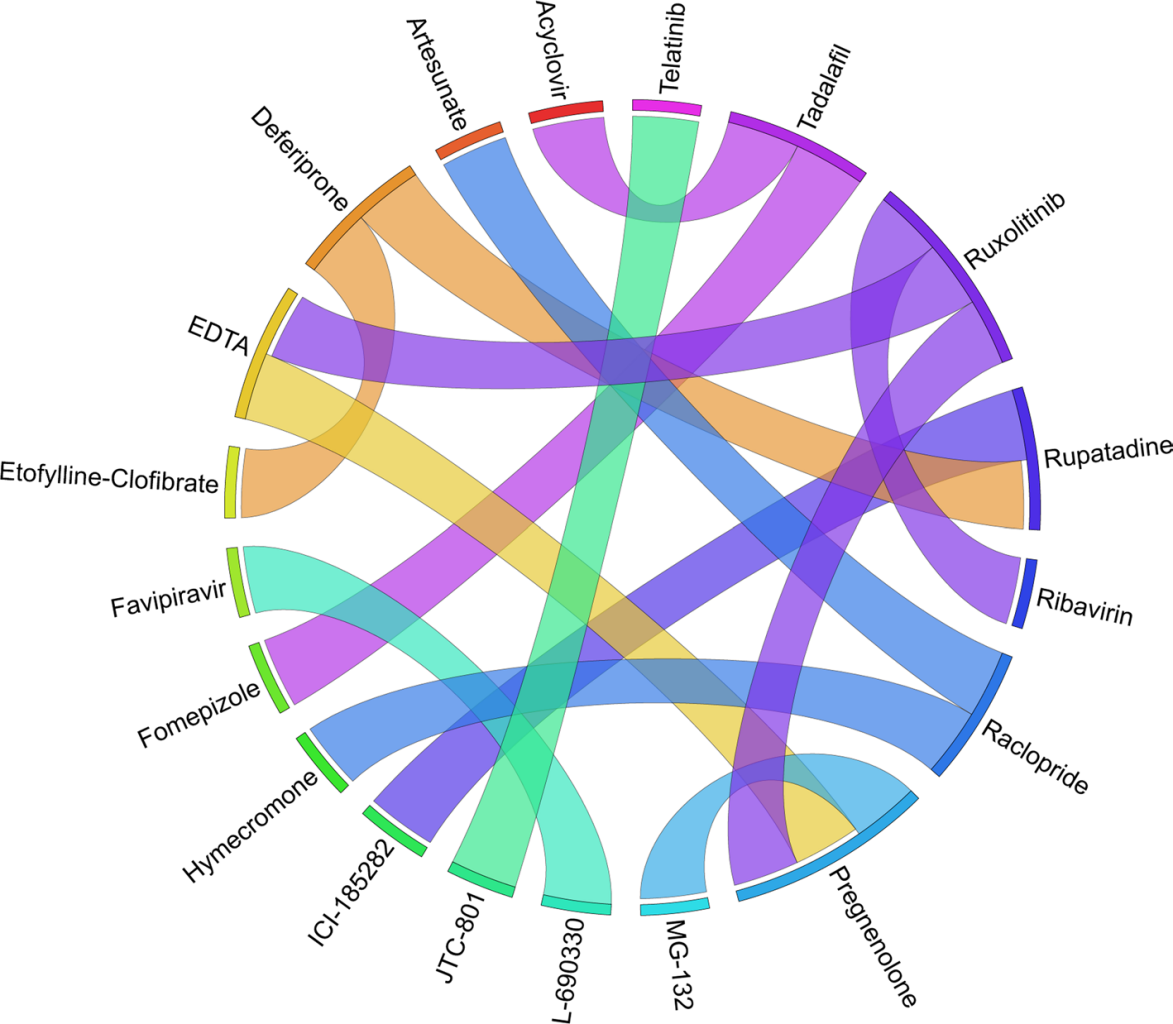
Top pairs of drugs with highest anti-correlation corresponding to subtype 3

**Fig. 9:**
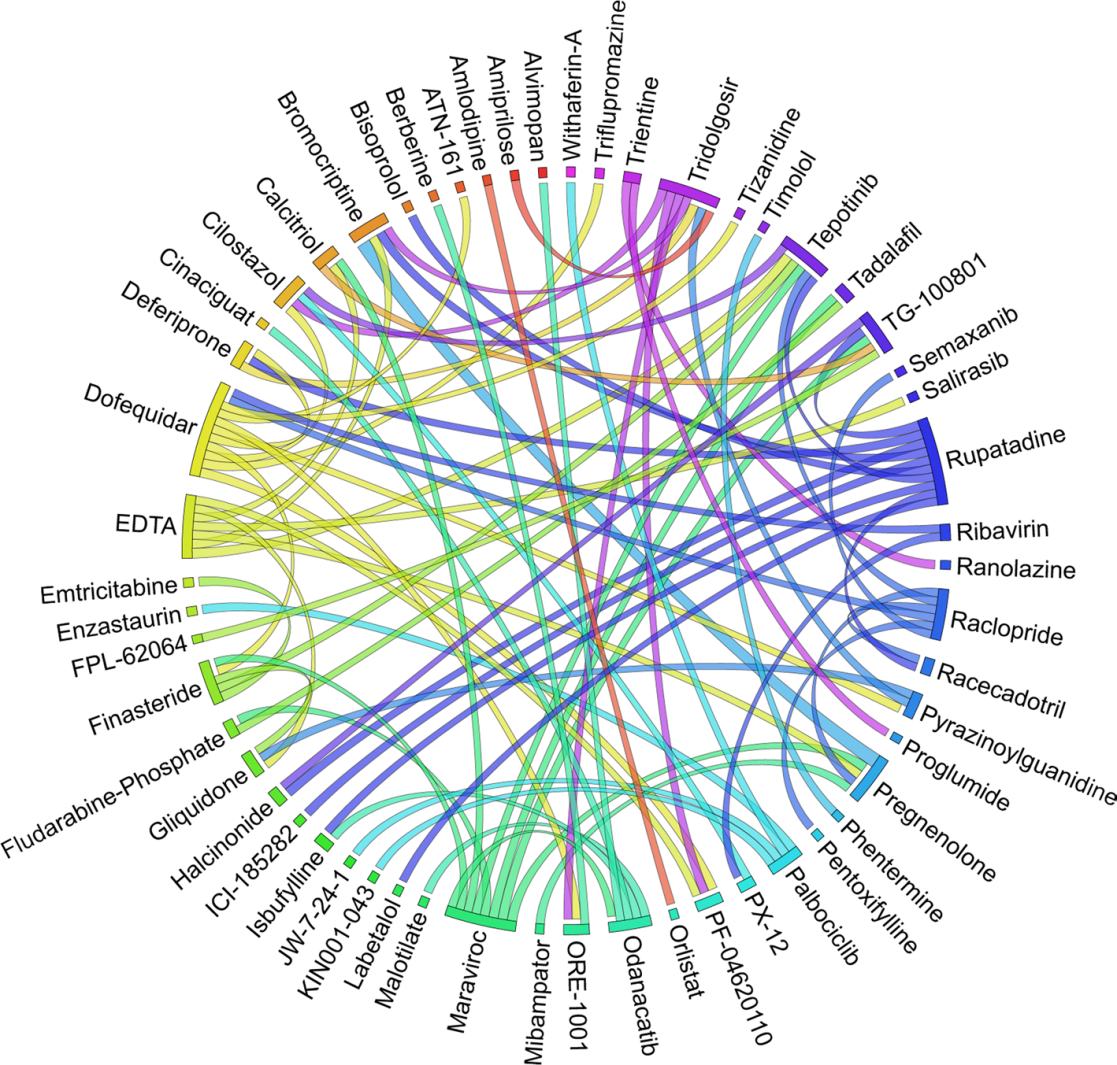
Top pairs of drugs with highest anti-correlation corresponding to subtype 4

**Fig. 10:**
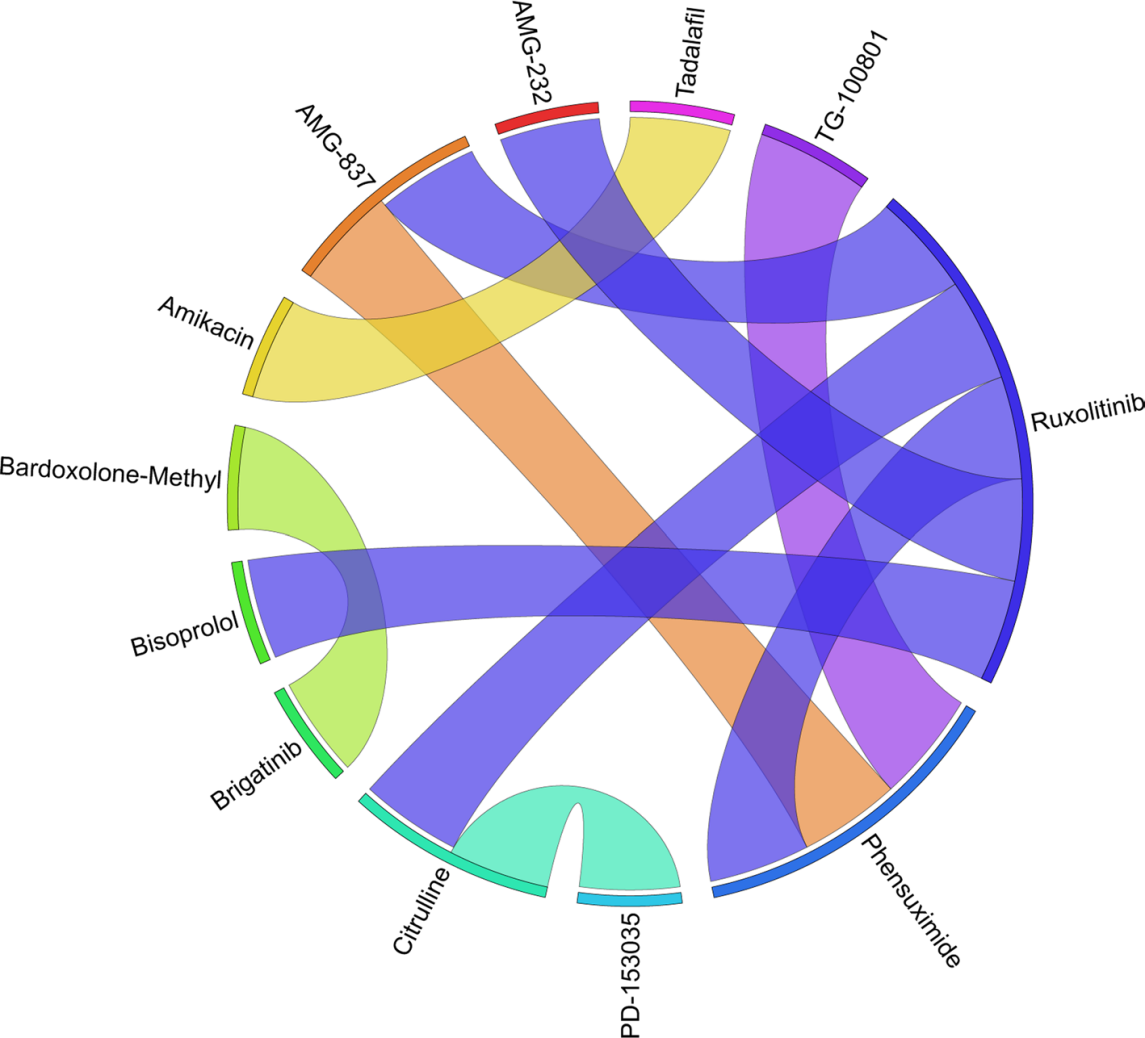
Top pairs of drugs with highest anti-correlation corresponding to subtype 5

**Fig. 11:**
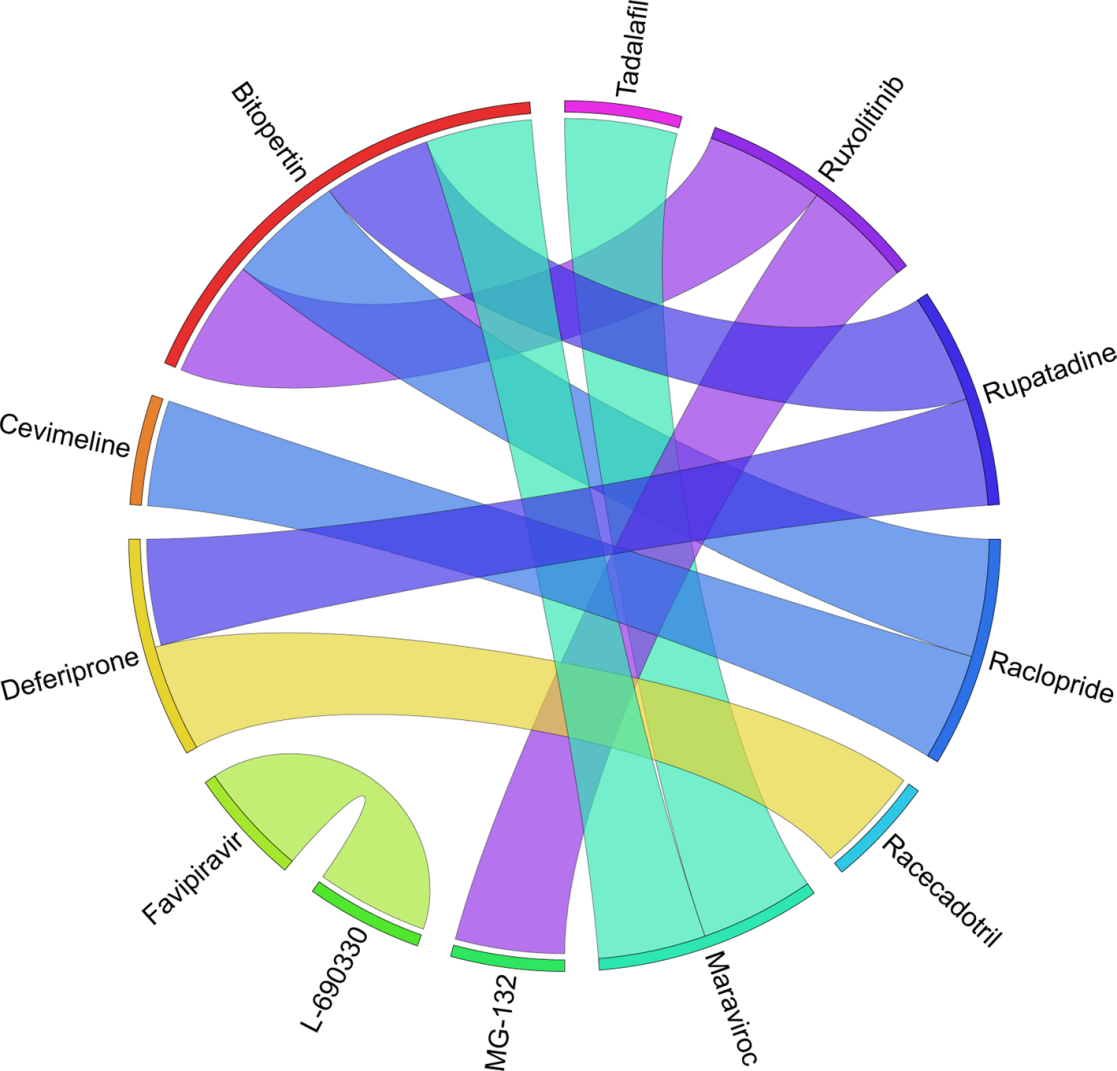
Top pairs of drugs with highest anti-correlation corresponding to subtype 6

**Fig. 12:**
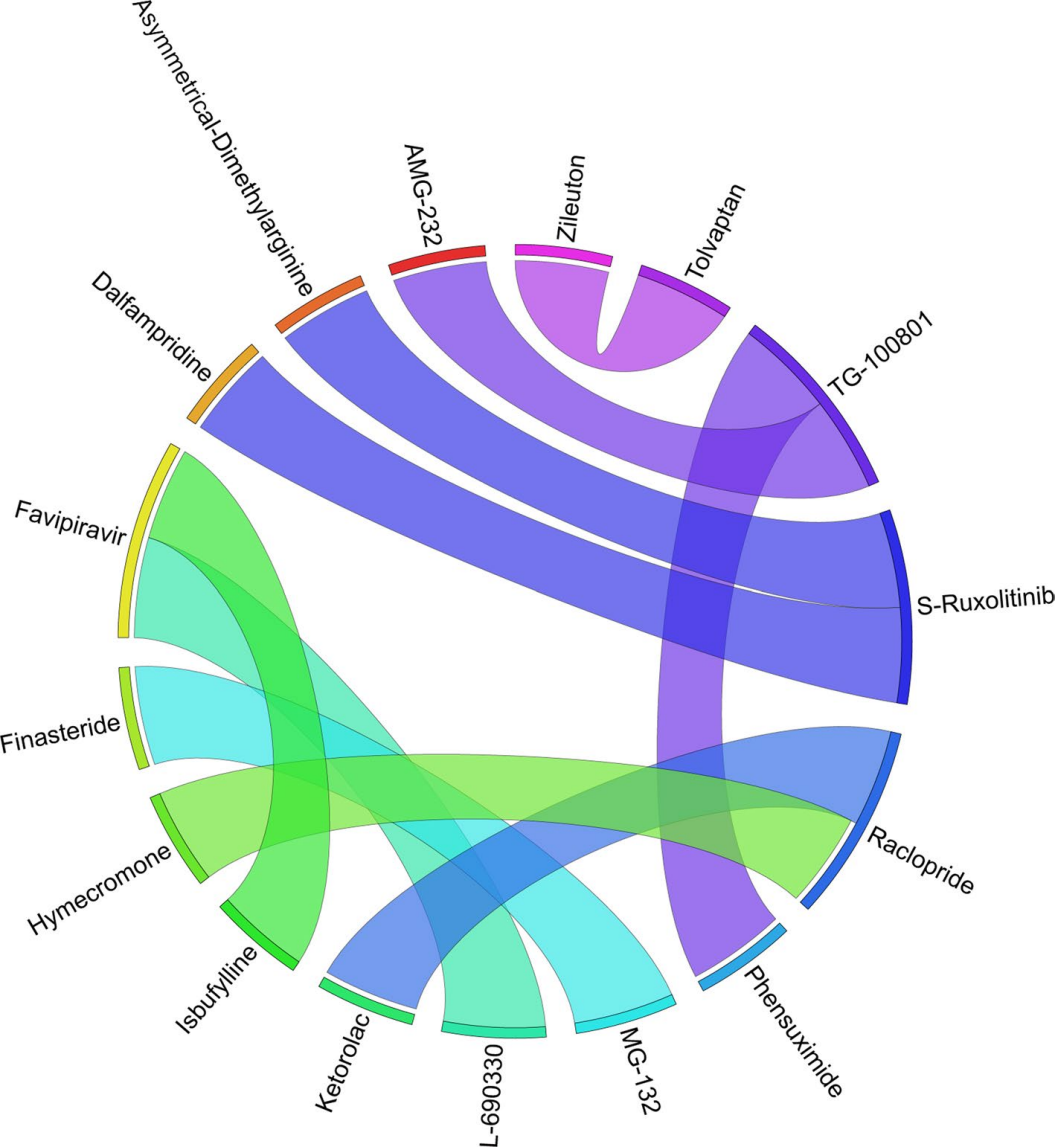
Top pairs of drugs with highest anti-correlation corresponding to subtype 7

**Fig. 13:**
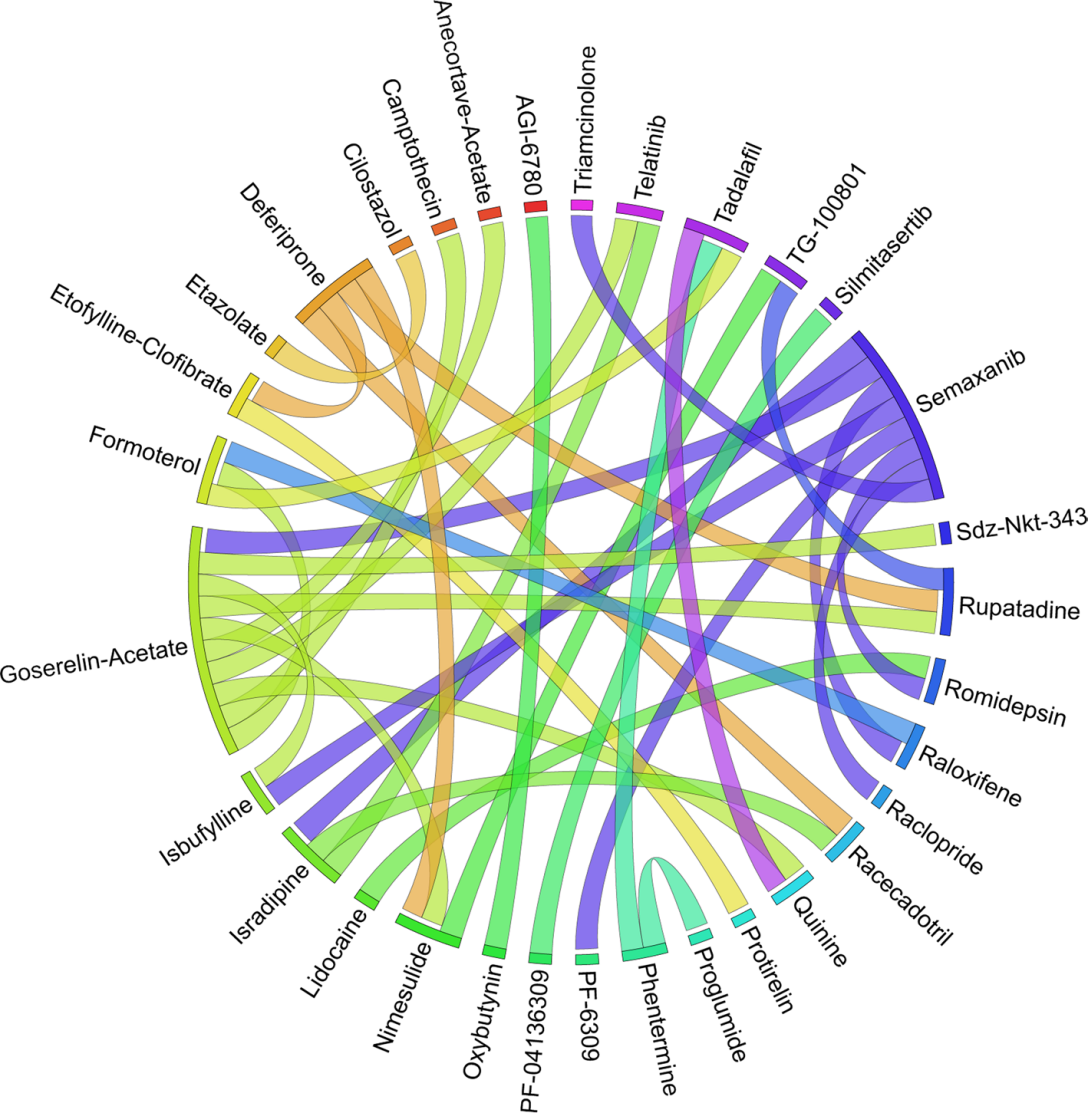
Top pairs of drugs with highest anti-correlation corresponding to subtype 8

**Fig. 14:**
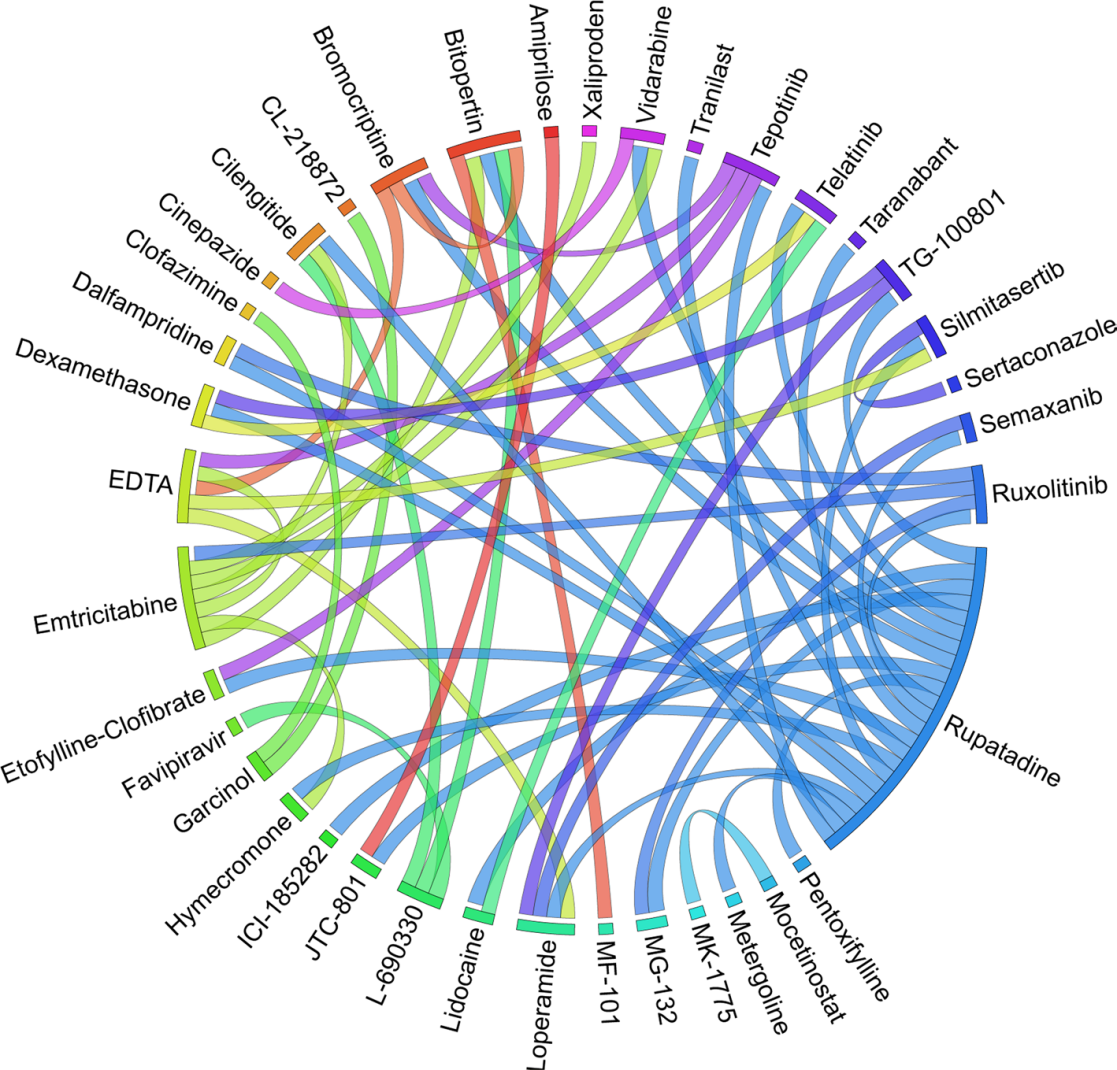
Top pairs of drugs with highest anti-correlation corresponding to subtype 9

**Fig. 15:**
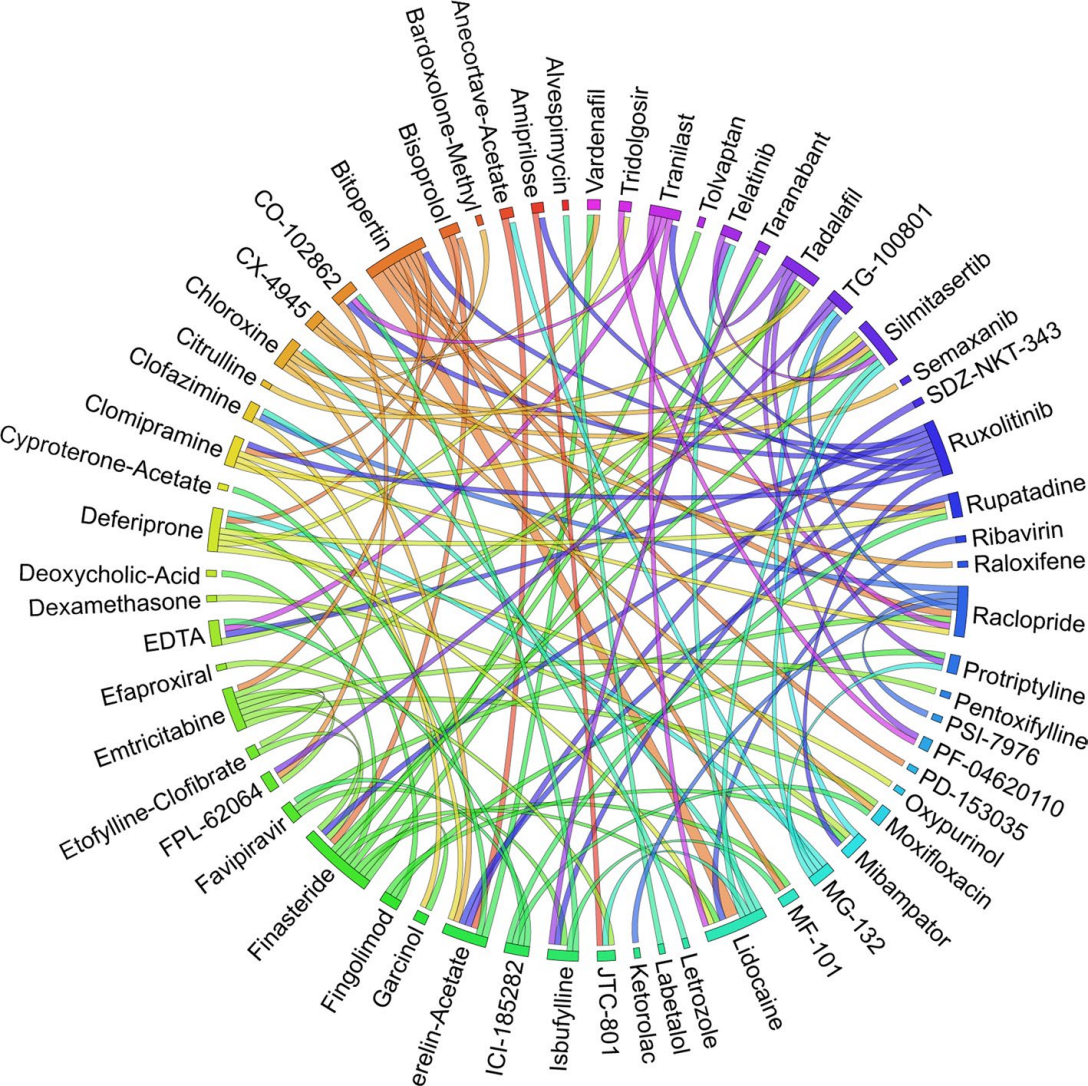
Top pairs of drugs with highest anti-correlation corresponding to subtype 10

Figures 6, 7, 8, 9, 10, 11, 12, 13, 14 and 15 depict the top pairs of drugs with the highest anti-correlation scores with Subtypes 1-10 of breast cancer. Here, we show the pairs of drugs that have a better anti-correlation score than the best single drug for each of these subtypes. Also, we limit the number of pairs to a maximum of 100 top pairs of such drugs, if there is more than 100 pairs with better score than the best single drug.

**Fig. 16:**
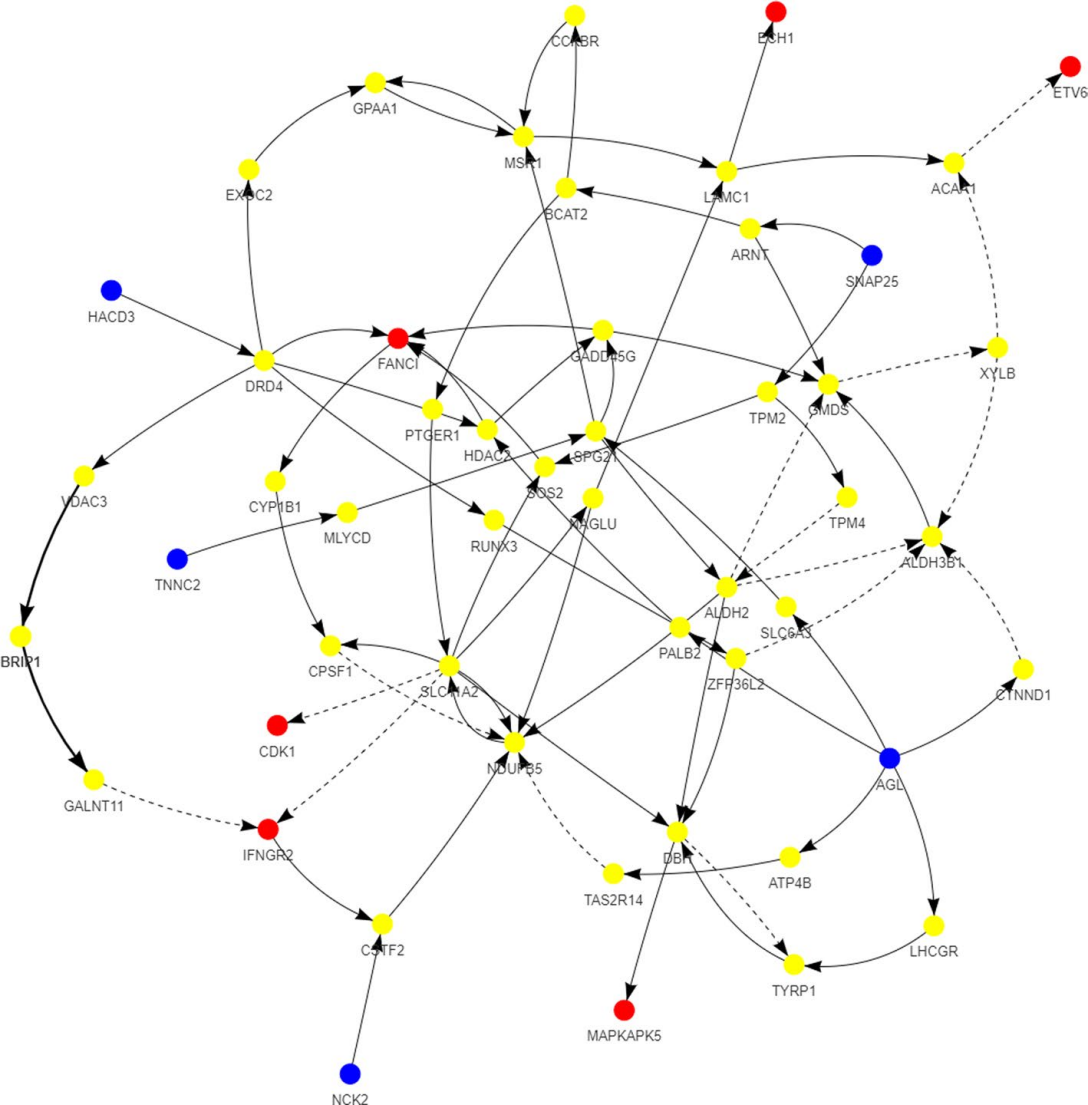
Drug-disease network (DDN) of Tadalafil and PF-04620110 drugs with subtype 1. Blue nodes depict drug related genes, while red nodes depict candidate genes related to subtype 1

Moreover, Tables 13, 14, 15, 16, 17, 18, 19, 20, 21 and 22 show top ten pairs of drugs for each of the ten breast cancer subtypes. Observing these tables, we infer that many of the top ranked pairs of drugs contain at least one individual top ranked drug, though there are some notable exceptions. For example, drugs *TG-100801* and *Phensuximide* are not even among the top 50 repurposed drugs corresponding to Subtype 2 when administered independently with mere correlation scores of − 0.57 and − 0.55 to subtype 2, respectively. However, when administered together, the correlation between that pair and subtype 2 grows to a noticeable − 0.97 range, which places the pair in the second spot among the top repurposed pairs for that subtype. We observe a similar catalyzing effect in combination of *Pregnenolone* and *Bromocriptine* with respect to subtype 4, and combination of *Amikacin* and *Tadalafil* with respect to subtype 5. Also, Fig. 16 depicts drug-disease network (DDN) of two perturbation agents (Tadalafil and PF-04620110) and subtype 1 of breast cancer. Blue nodes depict drug related genes, while red nodes depict candidate genes related to subtype 1. Also solid arrows depict activating relationship between involved genes, while dotted arrow depicts a suppressing relationship.

**Table 13:** Top 10 pairs of drugs with their correlation with subtype 1 of breast cancer, considering both combined and individually.

Drug1	Drug2	C. Cor.	Cor. 1	Cor. 2	C. Rank	Rank 1	Rank 2
Ruxolitinib	GSK-2636771	− 0.76	− 0.59	− 0.50	1	1	12
Tadalafil	PF-04620110	− 0.70	− 0.52	− 0.42	2	5	66
Deferiprone	Rimexolone	− 0.69	− 0.53	− 0.51	3	3	6
Deferiprone	Rupatadine	− 0.69	− 0.53	− 0.47	4	3	22
Raclopride	Racecadotril	− 0.69	− 0.47	− 0.45	5	24	35
Tranilast	GSK-2636771	− 0.68	− 0.52	− 0.50	6	4	12
Tadalafil	Amikacin	− 0.68	− 0.52	− 0.41	7	5	71
L-690330	Favipiravir	− 0.67	− 0.45	− 0.44	8	37	44
Deferiprone	Etofylline-Clofibrate	− 0.67	− 0.53	− 0.48	9	3	16
Ruxolitinib	Ribavirin	− 0.67	− 0.59	− 0.56	10	1	2

**Table 14:** Top 10 pairs of drugs with their correlation with subtype 2 of breast cancer, considering both combined and individually

Drug1	Drug2	C. Cor.	Cor. 1	Cor. 2	C. Rank	Rank 1	Rank 2
Goserelin-Acetate	Norethindrone	− 0.97	− 0.80	− 0.54	1	2	75
TG-100801	Phensuximide	− 0.97	− 0.57	− 0.55	2	50	64
Goserelin-acetate	Phensuximide	− 0.96	− 0.80	− 0.55	3	2	64
Goserelin-acetate	Nimesulide	− 0.96	− 0.80	− 0.54	4	2	66
L-690330	Favipiravir	− 0.94	− 0.71	− 0.53	5	7	83
Dopamine	Nimesulide	− 0.90	− 0.57	− 0.54	6	51	66
TG-100801	AMG-232	− 0.89	− 0.57	− 0.56	7	50	59
Tolvaptan	Zileuton	− 0.89	− 0.67	− 0.53	8	9	87
Emtricitabine	Finasteride	− 0.89	− 0.60	− 0.57	9	33	53
Nimesulide	Selamectin	− 0.89	− 0.54	− 0.54	10	66	72

**Table 15:** Top 10 pairs of drugs with their correlation with subtype 3 of breast cancer, considering both combined and individually

Drug1	Drug2	C. Cor.	Cor. 1	Cor. 2	C. Rank	Rank 1	Rank 2
Tadalafil	Acyclovir	− 0.79	− 0.61	− 0.45	1	4	73
Tadalafil	Fomepizole	− 0.78	− 0.61	− 0.43	2	4	100
Rupatadine	ICI-185282	− 0.77	− 0.58	− 0.52	3	6	21
Deferiprone	Etofylline-Clofibrate	− 0.76	− 0.62	− 0.57	4	3	9
Deferiprone	Rupatadine	− 0.75	− 0.62	− 0.58	5	3	6
Ruxolitinib	Ribavirin	− 0.74	− 0.68	− 0.57	6	2	7
L-690330	Favipiravir	− 0.74	− 0.49	− 0.47	7	38	54
Raclopride	Hymecromone	− 0.74	− 0.54	− 0.48	8	13	48
EDTA	Pregnenolone	− 0.74	− 0.56	− 0.51	9	10	28
Raclopride	Artesunate	− 0.74	− 0.54	− 0.47	10	13	59

**Table 16:** Top 10 pairs of drugs with their correlation with subtype 4 of breast cancer, considering both combined and individually

Drug1	Drug2	C. Cor.	Cor. 1	Cor. 2	C. Rank	Rank 1	Rank 2
Pregnenolone	Bromocriptine	− 0.99	− 0.42	− 0.40	1	24	38
EDTA	EMD-1214063	− 0.69	− 0.46	− 0.35	2	8	81
Maraviroc	Pregnenolone	− 0.68	− 0.46	− 0.42	3	9	24
Rupatadine	TG-100801	− 0.68	− 0.58	− 0.37	4	1	60
Maraviroc	TG-100801	− 0.67	− 0.46	− 0.37	5	9	60
Finasteride	Emtricitabine	− 0.66	− 0.43	− 0.37	6	19	68
Maraviroc	Tadalafil	− 0.66	− 0.46	− 0.35	7	9	89
Dofequidar	Triflupromazine	− 0.66	− 0.44	− 0.36	8	14	78
Dofequidar	PF-04620110	− 0.66	− 0.44	− 0.38	9	14	55
Dofequidar	Finasteride	− 0.65	− 0.44	− 0.43	10	14	19

**Table 17:** Top 10 pairs of drugs with their correlation with subtype 5 of breast cancer, considering both combined and individually

Drug1	Drug2	C. Cor.	Cor. 1	Cor. 2	C. Rank	Rank 1	Rank 2
TG-100801	Phensuximide	− 0.97	− 0.72	− 0.63	1	6	28
Ruxolitinib	Phensuximide	− 0.89	− 0.93	− 0.63	2	1	28
Ruxolitinib	AMG-837	− 0.89	− 0.93	− 0.83	3	1	2
AMG-837	Phensuximide	− 0.89	− 0.83	− 0.63	4	2	28
Ruxolitinib	Citrulline	− 0.89	− 0.93	− 0.82	5	1	3
Amikacin	Tadalafil	− 0.88	− 0.59	− 0.57	6	47	65
L-citrulline	PD-153035	− 0.88	− 0.82	− 0.62	7	3	35
Ruxolitinib	AMG-232	− 0.88	− 0.93	− 0.56	8	1	73
Bardoxolone-methyl	AP-26113	− 0.88	− 0.63	− 0.58	9	33	52
Ruxolitinib	Bisoprolol	− 0.88	− 0.93	− 0.71	10	1	8

**Table 18:** Top 10 pairs of drugs with their correlation with subtype 6 of breast cancer, considering both combined and individually

Drug1	Drug2	C. Cor.	Cor. 1	Cor. 2	C. Rank	Rank 1	Rank 2
Ruxolitinib	Bitopertin	− 0.73	− 0.67	− 0.40	1	1	44
Raclopride	Bitopertin	− 0.72	− 0.41	− 0.40	2	36	44
Maraviroc	Tadalafil	− 0.70	− 0.49	− 0.43	3	3	23
Favipiravir	L-690330	− 0.70	− 0.45	− 0.42	4	13	32
Ruxolitinib	MG-132	− 0.68	− 0.67	− 0.38	5	1	55
Rupatadine	Bitopertin	− 0.67	− 0.50	− 0.40	6	2	44
Maraviroc	Bitopertin	− 0.66	− 0.49	− 0.40	7	3	44
Raclopride	Cevimeline	− 0.66	− 0.41	− 0.36	8	36	74
Deferiprone	Racecadotril	− 0.66	− 0.49	− 0.48	9	4	8
Rupatadine	Deferiprone	− 0.66	− 0.50	− 0.49	10	2	4

**Table 19:** Top 10 pairs of drugs with their correlation with subtype 7 of breast cancer, considering both combined and individually

Drug1	Drug2	C. Cor.	Cor. 1	Cor. 2	C. Rank	Rank 1	Rank 2
L-690330	Favipiravir	− 0.93	− 0.69	− 0.54	1	12	87
MG-132	Finasteride	− 0.92	− 0.66	− 0.55	2	18	83
TG-100801	Phensuximide	− 0.91	− 0.69	− 0.61	3	11	37
Raclopride	Ketorolac	− 0.89	− 0.65	− 0.57	4	19	57
Hymecromone	Raclopride	− 0.88	− 0.66	− 0.65	5	16	19
Ruxolitinib	ADMA	− 0.87	− 0.83	− 0.57	6	2	56
Ruxolitinib	Dalfampridine	− 0.87	− 0.83	− 0.63	7	2	24
TG-100801	AMG-232	− 0.87	− 0.69	− 0.59	8	11	51
Tolvaptan	Zileuton	− 0.87	− − 0.64	− 0.59	9	22	49
Isbufylline	Favipiravir	− 0.86	− 0.71	− 0.54	10	7	87

**Table 20:** Top 10 pairs of drugs with their correlation with subtype 8 of breast cancer, considering both combined and individually

Drug1	Drug2	C. Cor.	Cor. 1	Cor. 2	C. Rank	Rank 1	Rank 2
Emaxanib	Isradipine	− 0.71	− 0.60	− 0.42	1	1	34
Semaxanib	Goserelin-Acetate	− 0.68	− 0.60	− 0.55	2	1	3
Goserelin-Acetate	Formoterol	− 0.68	− 0.55	− 0.43	3	3	31
Goserelin-Acetate	Camptothecin	− 0.66	− 0.55	− 0.38	4	3	72
Deferiprone	Racecadotril	− 0.66	− 0.49	− 0.40	5	6	56
Deferiprone	Etofylline-Clofibrate	− 0.65	− 0.49	− 0.47	6	6	15
Semaxanib	Raloxifene	− 0.65	− 0.60	− 0.54	7	1	4
Lidocaine	Romidepsin	− 0.64	− 0.46	− 0.41	8	17	49
Goserelin-Acetate	Telatinib	− 0.64	− 0.55	− 0.41	9	3	44
Isradipine	Telatinib	− 0.64	− 0.42	− 0.41	10	34	44

**Table 21:** Top 10 pairs of drugs with their correlation with subtype 9 of breast cancer, considering both combined and individually

Drug1	Drug2	C. Cor.	Cor. 1	Cor. 2	C. Rank	Rank 1	Rank 2
Rupatadine	TG-100801	− 0.85	− 0.72	− 0.61	1	1	8
L-690330	Favipiravir	− 0.83	− 0.65	− 0.48	2	5	76
Rupatadine	Lidocaine	− 0.82	− 0.72	− 0.51	3	1	51
Rupatadine	MG-132	− 0.82	− 0.72	− 0.61	4	1	9
Rupatadine	ICI-185,282	− 0.81	− 0.72	− 0.52	5	1	35
Rupatadine	Telatinib	− 0.79	− 0.72	− 0.47	6	1	82
Rupatadine	Vidarabine	− 0.79	− 0.72	− 0.59	7	1	12
Garcinol	CL-218872	− 0.78	− 0.56	− 0.49	8	24	60
Rupatadine	Bitopertin	− 0.77	− 0.72	− 0.48	9	1	71
Emtricitabine	Bitopertin	− 0.77	− 0.65	− 0.48	10	4	71

**Table 22:** Top 10 pairs of drugs with their correlation with subtype 10 of breast cancer, considering both combined and individually

Drug1	Drug2	C. Cor.	Cor. 1	Cor. 2	C. Rank	Rank 1	Rank 2
Tadalafil	Telatinib	− 0.86	− 0.55	− 0.53	1	47	61
Bitopertin	Lidocaine	− 0.85	− 0.66	− 0.57	2	2	35
Anecortave-Acetate	Goserelin-Acetate	− 0.84	− 0.56	− 0.52	3	38	64
Finasteride	Tadalafil	− 0.84	− 0.60	− 0.55	4	16	47
Bitopertin	Raclopride	− 0.83	− 0.66	− 0.57	5	2	36
SDZ-NKT-343	Goserelin-acetate	− 0.83	− 0.59	− 0.52	6	24	64
Goserelin-Acetate	Vardenafil	− 0.81	− 0.52	− 0.48	7	64	96
Favipiravir	Isbufylline	− 0.81	− 0.59	− 0.58	8	25	28
Bisoprolol	Finasteride	− 0.80	− 0.61	− 0.60	9	12	16
Finasteride	Garcinol	− 0.80	− 0.60	− 0.55	10	16	48

*Goserelin-Acetate*, which is sold under brand name *Zoladex* among others, is as a sex hormone suppression drug approved by FDA intended for use in the treatment of breast and prostate cancer [61]. As shown in Tables 3,14, *Goserelin-Acetate* as a single drug produces an correlation score of -0.8 with respect to subtype 2 of BC, which places it in the top spot among the single drugs for this subtype. However, if combined with either *Norethindrone*, *Phensuximide* or *Nimesulide*, the correlation score decreases to almost -0.97. This means that combining either of the aforementioned drugs with *Goserelin-Acetate* can result in a more effective therapeutic drug for this BC subtype.

### Triple negative breast cancer subtype

Table 23 shows the identified driver genes corresponding to the TN group. Moreover, Table 24 shows the top 20 single repurposed drugs for the triple negative breast cancer (TNBC) subtype. As shown in the table, *Ruxolitinib* is by far the most negatively correlated drug for TNBC subtype and can be investigated further for its effectiveness on this particular type of breast cancer [51, 52, 62].

**Table 23:** Identified driver genes associated with triple negative breast cancer subtype

ACRV1	ADCY9	AKT2	ALOX12B	APTX	C17orf100	C1QBP	CALM2
CARD18	CLPSL1	DARS2	DEFB136	DHX33	EEF1E1	EHHADH	ELAC2
EPPIN	FAXDC2	FOXO3	GAB2	GAL3ST3	GFER	GJA10	GUCA2A
HACE1	HP	HTR3D	IFNA21	KLHDC8A	LDOC1L	LINC00628	LINC00919
MFAP4	MPRIP	MRGPRF	MRPL13	MUC21	OR1S1	OR3A1	PLEKHA8
PMCHL1	PNPLA3	POGK	POLR3G	PRPH2	RFPL4B	SDHC	SIRT5
SLC1A4	SLC25A11	SLC35F2	SLFN12L	SNX29	SRPK1	STOML2	SUV39H2
TAS2R20	TATDN1	THOC1	TOMM22	TRIM72	TRMT12	TWIST2	TXNDC17
URB2	VDAC3	WFDC10A	ZC3H7B	ZNF23

**Table 24:** Top 20 drugs along with their ranking corresponding to Triple negative breast cancer subtype

Rank	Drug Name	Pearson Correlation	Dosage	Time (h)
1	Ruxolitinib	− 0.765	10	24
2	Raloxifene	− 0.652	0.04	24
3	PF-04217903	− 0.649	0.04	24
4	PD-173074	− 0.603	10	24
5	TG-100801	− 0.591	0.04	24
6	Dexamethasone	− 0.589	0.04	24
7	Semaxanib	− 0.588	0.04	24
8	Rupatadine	− 0.575	0.04	24
9	Tranilast	− 0.574	0.04	24
10	Bromocriptine	− 0.565	0.04	24
11	ICI-185282	− 0.562	0.04	24
12	Hymecromone	− 0.558	0.04	24
13	Emtricitabine	− 0.556	10	24
14	Phentermine	− 0.554	0.04	24
15	Fludarabine-Phosphate	− 0.548	10	24
16	JTC-801	− 0.547	0.04	24
17	AMG-232	− 0.541	10	24
18	Lidocaine	− 0.525	0.04	24
19	Amiprilose	− 0.520	0.04	24
20	Labetalol	− 0.515	0.04	24

**Fig. 17:**
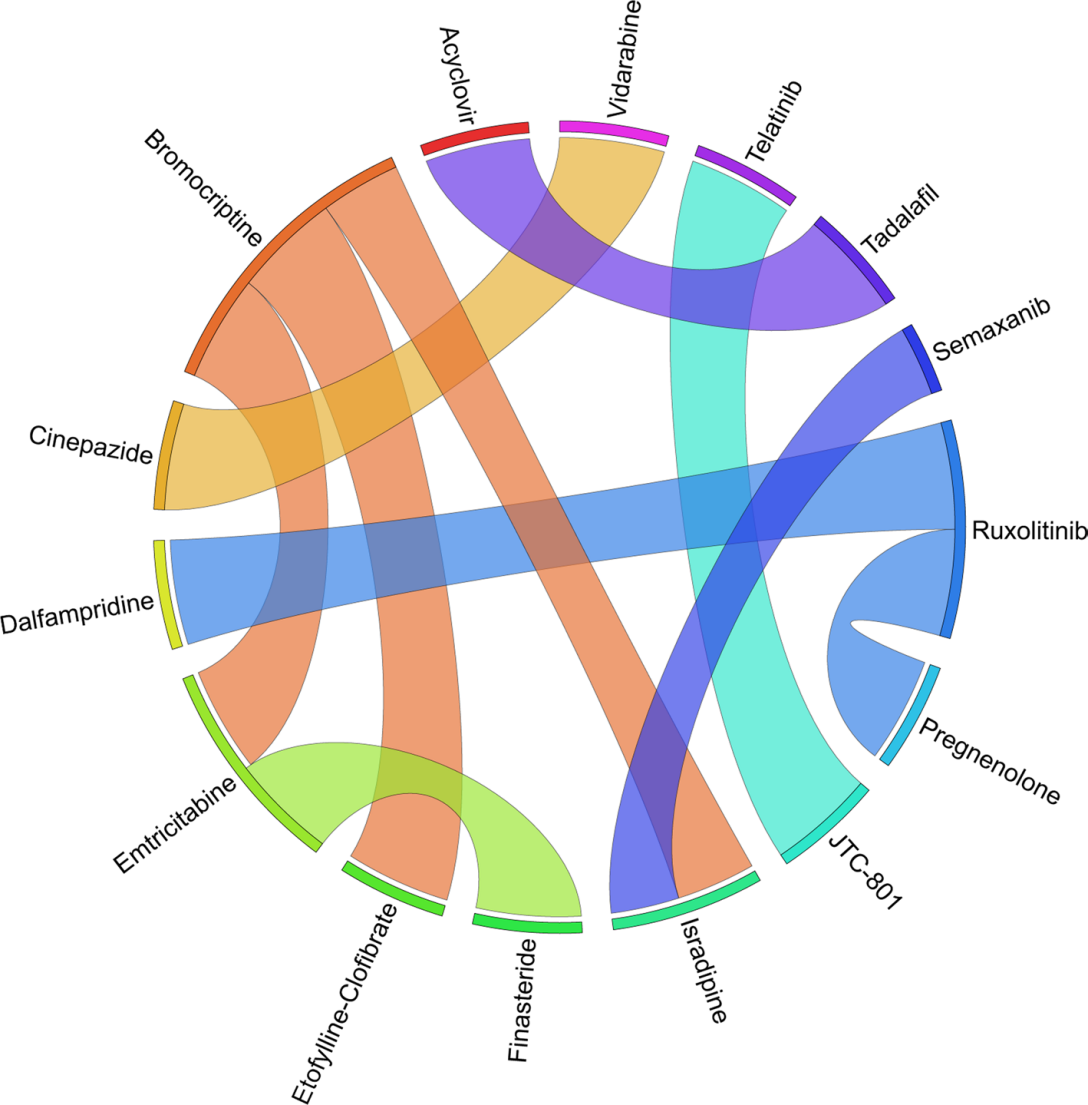
Top pairs of drugs with highest anti-correlation corresponding to TNBC subtype

Moreover, Table 25 and Fig. 17 show the top 10 pairs of repurposed drugs and their corresponding scores with respect to TNBC subtype. We observe that despite being the tenth repurposed single drug, *Bromocriptine* is managed to become one of the most effective repurposed drugs when paired with *Isradipin*, *Emtricitabine* and *Etofylline-Clofibrate*. Although *Bromocriptine* has been suggested in earlier studies as a potential repurposed drug in cancer therapy [63], these new combinations have not seem to be evaluated so far for breast cancer treatment, which can be investigated further both computationally and clinically. Another interesting observation from Tables 24, 25 is that there are only four pairs of drugs with anti-correlation scores better than *Ruxolitinib* as the best single drug identified for TNBC subtype.

**Table 25:** Top 10 pairs of drugs with their correlation with respect to TN breast cancer subtype, both when they are combined and individually

Drug1	Drug2	C. Cor.	Cor. 1	Cor. 2	C. Rank	Rank 1	Rank 2
Bromocriptine	Isradipine	− 0.85	− 0.57	− 0.47	1	10	51
Cinepazide	Vidarabine	− 0.80	− 0.45	− 0.45	2	74	75
Bromocriptine	Emtricitabine	− 0.79	− 0.57	− 0.56	3	10	13
Bromocriptine	Etofylline-Clofibrate	− 0.77	− 0.57	− 0.51	4	10	22
Emtricitabine	Finasteride	− 0.76	− 0.56	− 0.50	5	13	26
JTC-801	Telatinib	− 0.75	− 0.55	− 0.47	6	16	47
Ruxolitinib	Dalfampridine	− 0.74	− 0.77	− 0.50	7	1	31
Semaxanib	Isradipine	− 0.74	− 0.59	− 0.47	8	7	51
Ruxolitinib	Pregnenolone	− 0.74	− 0.77	− 0.48	9	1	40
Tadalafil	Acyclovir	− 0.74	− 0.47	− 0.46	10	43	58

Based on these observations, we infer that from both single drug and paired drug experiments, there are some promising drugs that can be repurposed either individually or in combination with another drug (as a pair) with potential therapeutic effects for each of the ten breast cancer subtypes. Some of these drugs such as *Ruxolitinib* have a high anti-correlation score for most of the subtypes, while some of the drugs such as *Maraviroc* [64] seem to be more effective on a particular subtype rather than on others. The fact that the top single drug, *Ruxolitinib*, is currently in multiple clinical trials in patients with metastatic breast cancer [51, 56] shows that the proposed method is able to computationally predict the potential therapeutic effect of this drug on multiple breast cancer subtypes, as well as on TNBC subtype. Indeed, further wet lab analysis is needed to determine the therapeutic level of identified drugs on each breast cancer subtype.

## Conclusion and future work

The proposed computational drug repurposing method is a network-based integration approach that combines transcriptomic, genomics and pathway data in order to find computationally promising repurposed single or paired drugs for each breast cancer subtype as well as TNBC subtype. Some of the top identified drugs are either known (breast) cancer drugs or in different trial phases to be repurposed for breast or other types of cancer, while some of the identified single or paired drugs have not been used for breast cancer treatment yet, which provides opportunity for further clinical experiments and trials. Using genomic and transcriptomic data as well as both copy number variations and copy number aberrations would help the initial process to identify the driver genes more effectively and hence finding the final set of repourposed drugs that can be highly effective for treatment of other types of cancer.

The proposed framework has the potential of identifying a combination of more than two drugs, which could help identifying new and enhanced sets of drugs for various types of cancer as well as other types of diseases. Also as a possible future work, the framework can be extended to leverage more complex and nonlinear combination of drugs in order to find the most suitable sets of drugs for each disease. Moreover, since for the simplicity of the process we excluded pairs of drugs with known drug-drug interference, potential drug-drug interactions have not been considered yet. So, another possible future work could be extending the framework to include such pairs of drugs with known interactions and their effect on the disease.

## Data Availability

The dataset analysed during the current study are available in the GEO NCBI repository, https://www.ncbi.nlm.nih.gov/geo/query/acc.cgi?acc=GSE70138

## References

[1] Sertkaya A, Birkenbach A, Berlind A, Eyraud J. Examination of clinical trial costs and barriers for drug development. Report, US Department of Health and Human Services, Office of the Assistant Secretary for Planning and Evaluation, Washington, DC; 2014. p. 1–92.

[2] Adams CP, Brantner VV (2006). Estimating the cost of new drug development: is it really $802 million?. Health Aff.

[3] Dickson M, Gagnon JP (2009). The cost of new drug discovery and development. Discov Med.

[4] Deotarse P, Jain A, Baile M, Kolhe N, Kulkarni A (2015). Drug repositioning: a review. Int J Pharm Res Rev.

[5] Wong CH, Siah KW, Lo AW (2019). Estimation of clinical trial success rates and related parameters. Biostatistics.

[6] DiMasi JA, Feldman L, Seckler A, Wilson A (2010). Trends in risks associated with new drug development: success rates for investigational drugs. Clin Pharmacol Ther.

[7] Oprea TI, Overington JP (2015). Computational and practical aspects of drug repositioning. Assay Drug Dev Technol.

[8] Napolitano F, Zhao Y, Moreira VM, Tagliaferri R, Kere J, D’Amato M, Greco D (2013). Drug repositioning: a machine-learning approach through data integration. J Cheminform.

[9] Lotfi Shahreza M, Ghadiri N, Mousavi SR, Varshosaz J, Green JR (2017). A review of network-based approaches to drug repositioning. Brief Bioinform.

[10] Gönen M (2012). Predicting drug-target interactions from chemical and genomic kernels using Bayesian matrix factorization. Bioinformatics.

[11] Zou J, Zheng M-W, Li G, Su Z-G (2013). Advanced systems biology methods in drug discovery and translational biomedicine. BioMed Res Int.

[12] Xue H, Li J, Xie H, Wang Y (2018). Review of drug repositioning approaches and resources. Int J Biol Sci.

[13] Luo H, Li M, Wang S, Liu Q, Li Y, Wang J (2018). Computational drug repositioning using low-rank matrix approximation and randomized algorithms. Bioinformatics.

[14] Zhang P, Wang F, Hu J. Towards drug repositioning: a unified computational framework for integrating multiple aspects of drug similarity and disease similarity. In: AMIA annual symposium proceedings, 2014. American Medical Informatics Association; 2014. p. 1258. PMC4419869 25954437

[15] Li J, Zhu X, Chen JY (2009). Building disease-specific drug-protein connectivity maps from molecular interaction networks and PubMed abstracts. PLoS Comput Biol.

[16] Gramatica R, Di Matteo T, Giorgetti S, Barbiani M, Bevec D, Aste T (2014). Graph theory enables drug repurposing—how a mathematical model can drive the discovery of hidden mechanisms of action. PLoS ONE.

[17] Rastegar-Mojarad M, Elayavilli RK, Li D, Prasad R, Liu H. A new method for prioritizing drug repositioning candidates extracted by literature-based discovery. In: 2015 IEEE international conference on bioinformatics and biomedicine (BIBM). IEEE; 2015. p. 669–74

[18] Jang G, Lee T, Lee BM, Yoon Y (2017). Literature-based prediction of novel drug indications considering relationships between entities. Mol BioSyst.

[19] Kuusisto F, Steill J, Kuang Z, Thomson J, Page D, Stewart R (2017). A simple text mining approach for ranking pairwise associations in biomedical applications. AMIA Jt Summits Transl Sci Proc.

[20] Zhang M, Schmitt-Ulms G, Sato C, Xi Z, Zhang Y, Zhou Y, St George-Hyslop P, Rogaeva E (2016). Drug repositioning for Alzheimer’s disease based on systematic ‘omics’ data mining. PLoS ONE.

[21] Krallinger M, Erhardt RA-A, Valencia A (2005). Text-mining approaches in molecular biology and biomedicine. Drug Discov Today.

[22] Palma G, Vidal M-E, Raschid L. Drug-target interaction prediction using semantic similarity and edge partitioning. In: International semantic web conference. Springer; 2014. p. 131–46.

[23] Mullen J, Cockell SJ, Woollard P, Wipat A (2016). An integrated data driven approach to drug repositioning using gene-disease associations. PLoS ONE.

[24] Zhu Q, Tao C, Shen F, Chute CG (2014). Exploring the pharmacogenomics knowledge base (PharmGKB) for repositioning breast cancer drugs by leveraging web ontology language (OWL) and cheminformatics approaches. Biocomputing.

[25] Wu H, Gao L, Dong J, Yang X (2014). Detecting overlapping protein complexes by rough-fuzzy clustering in protein-protein interaction networks. PLoS ONE.

[26] Yu L, Huang J, Ma Z, Zhang J, Zou Y, Gao L (2015). Inferring drug-disease associations based on known protein complexes. BMC Med Genom.

[27] Wu C, Gudivada RC, Aronow BJ, Jegga AG (2013). Computational drug repositioning through heterogeneous network clustering. BMC Syst Biol.

[28] Šubelj L, Bajec M (2011). Unfolding communities in large complex networks: combining defensive and offensive label propagation for core extraction. Phys Rev E.

[29] Emig D, Ivliev A, Pustovalova O, Lancashire L, Bureeva S, Nikolsky Y, Bessarabova M (2013). Drug target prediction and repositioning using an integrated network-based approach. PLoS ONE.

[30] Köhler S, Bauer S, Horn D, Robinson PN (2008). Walking the interactome for prioritization of candidate disease genes. Am J Human Genet.

[31] Vanunu O, Magger O, Ruppin E, Shlomi T, Sharan R (2010). Associating genes and protein complexes with disease via network propagation. PLoS Comput Biol.

[32] Martínez V, Navarro C, Cano C, Fajardo W, Blanco A (2015). DrugNet: network-based drug–disease prioritization by integrating heterogeneous data. Artif Intell Med.

[33] Sun Y, Sheng Z, Ma C, Tang K, Zhu R, Wu Z, Shen R, Feng J, Wu D, Huang D (2015). Combining genomic and network characteristics for extended capability in predicting synergistic drugs for cancer. Nat Commun.

[34] Peyvandipour A, Saberian N, Shafi A, Donato M, Draghici S (2018). A novel computational approach for drug repurposing using systems biology. Bioinformatics.

[35] Sun M, Zhao S, Gilvary C, Elemento O, Zhou J, Wang F (2020). Graph convolutional networks for computational drug development and discovery. Brief Bioinform.

[36] Bourdakou MM, Athanasiadis EI, Spyrou GM (2016). Discovering gene re-ranking efficiency and conserved gene-gene relationships derived from gene co-expression network analysis on breast cancer data. Sci Rep.

[37] Curtis C, Shah SP, Chin S-F, Turashvili G, Rueda OM, Dunning MJ, Speed D, Lynch AG, Samarajiwa S, Yuan Y (2012). The genomic and transcriptomic architecture of 2,000 breast tumours reveals novel subgroups. Nature.

[38] McCann KE, Hurvitz SA, McAndrew N. Advances in targeted therapies for triple-negative breast cancer. Drugs. 2019;1–14. 10.1007/s40265-019-01155-4 31254268

[39] Beroukhim R, Getz G, Nghiemphu L, Barretina J, Hsueh T, Linhart D, Vivanco I, Lee JC, Huang JH, Alexander S (2007). Assessing the significance of chromosomal aberrations in cancer: methodology and application to glioma. Proc Natl Acad Sci.

[40] Raymond M, Rousset F (1995). An exact test for population differentiation. Evolution.

[41] International HapMap 3 Consortium (2010). Integrating common and rare genetic variation in diverse human populations. Nature.

[42] Liu H, Setiono R. Chi2: feature selection and discretization of numeric attributes. In: 2012 IEEE 24th international conference on tools with artificial intelligence. IEEE Computer Society; 1995. p. 388.

[43] Thomas JG, Olson JM, Tapscott SJ, Zhao LP (2001). An efficient and robust statistical modeling approach to discover differentially expressed genes using genomic expression profiles. Genome Res.

[44] Kanehisa M, Furumichi M, Sato Y, Ishiguro-Watanabe M, Tanabe M (2021). KEGG: integrating viruses and cellular organisms. Nucleic Acids Res.

[45] Biological Pathways Fact Sheet; 2022. https://www.genome.gov/about-genomics/fact-sheets/Biological-Pathways-Fact-Sheet.

[46] Firoozbakht F, Rezaeian I, D’agnillo M, Porter L, Rueda L, Ngom A (2017). An integrative approach for identifying network biomarkers of breast cancer subtypes using genomic, interactomic, and transcriptomic data. J Comput Biol.

[47] Lamb J, Crawford ED, Peck D, Modell JW, Blat IC, Wrobel MJ, Lerner J, Brunet J-P, Subramanian A, Ross KN (2006). The connectivity map: using gene-expression signatures to connect small molecules, genes, and disease. Science.

[48] Sirota M, Dudley JT, Kim J, Chiang AP, Morgan AA, Sweet-Cordero A, Sage J, Butte AJ (2011). Discovery and preclinical validation of drug indications using compendia of public gene expression data. Sci Transl Med.

[49] Palbociclib and Tadalafil Interactions. https://www.drugs.com/drug-interactions/palbociclib-with-tadalafil-3602-0-2144-0.html.

[50] Drugs approved for breast cancer. https://www.cancer.gov/about-cancer/treatment/drugs/breast. Last accessed on 15 Aug 2020.

[51] Stover DG, Del Alcazar CRG, Brock J, Guo H, Overmoyer B, Balko J, Xu Q, Bardia A, Tolaney SM, Gelman R (2018). Phase II study of ruxolitinib, a selective JAK1/2 inhibitor, in patients with metastatic triple-negative breast cancer. NPJ Breast Cancer.

[52] Kim JW, Gautam J, Kim JE, Kim J, Kang KW (2019). Inhibition of tumor growth and angiogenesis of tamoxifen-resistant breast cancer cells by ruxolitinib, a selective JAK2 inhibitor. Oncol Lett.

[53] Pinsky PF, Miller EA, Heckman-Stoddard BM, Minasian L (2020). Breast cancer characteristics and survival among users versus nonusers of raloxifene. Cancer Prev Res.

[54] Ağardan NM, Değim Z, Yılmaz Ş, Altıntaş L, Topal T (2020). Tamoxifen/raloxifene loaded liposomes for oral treatment of breast cancer. J Drug Deliv Sci Technol.

[55] Mesa RA, Yasothan U, Kirkpatrick P (2012). Ruxolitinib. Nat Rev Drug Discov.

[56] Lynce F, Williams JT, Regan MM, Bunnell CA, Freedman RA, Tolaney SM, Chen WY, Mayer EL, Partridge AH, Winer EP (2021). Phase I study of JAK1/2 inhibitor ruxolitinib with weekly paclitaxel for the treatment of HER2-negative metastatic breast cancer. Cancer Chemother Pharmacol.

[57] Brasca MG, Albanese C, Alzani R, Amici R, Avanzi N, Ballinari D, Bischoff J, Borghi D, Casale E, Croci V (2010). Optimization of 6, 6-dimethyl pyrrolo [3, 4-c] pyrazoles: identification of PHA-793887, a potent CDK inhibitor suitable for intravenous dosing. Bioorg Med Chem.

[58] Dextras C, Dashnyam M, Griner LAM, Sundaresan J, Chim B, Yu Z, Vodnala S, Lee C-CR, Hu X, Southall N (2020). Identification of small molecule enhancers of immunotherapy for melanoma. Sci Rep.

[59] PHA-793887|Drug Bank. https://go.drugbank.com/drugs/DB12686. Accessed: 20 Feb 2021.

[60] Wehde BL, Rädler PD, Shrestha H, Johnson SJ, Triplett AA, Wagner K-U (2018). Janus kinase 1 plays a critical role in mammary cancer progression. Cell Rep.

[61] Leo CP, Hentschel B, Szucs TD, Leo C (2020). FDA and EMA approvals of new breast cancer drugs—a comparative regulatory analysis. Cancers.

[62] Li W, Yang H, Li X, Han L, Xu N, Shi A (2019). Signaling pathway inhibitors target breast cancer stem cells in triple-negative breast cancer. Oncol Rep.

[63] Seo E-J, Sugimoto Y, Greten HJ, Efferth T (2018). Repurposing of bromocriptine for cancer therapy. Front Pharmacol.

[64] Pervaiz A, Zepp M, Mahmood S, Ali DM, Berger MR, Adwan H (2019). CCR5 blockage by maraviroc: a potential therapeutic option for metastatic breast cancer. Cell Oncol.

[65] Casaos J, Gorelick NL, Huq S, Choi J, Xia Y, Serra R, Felder R, Lott T, Kast RE, Suk I (2019). The use of ribavirin as an anticancer therapeutic: will it go viral?. Mol Cancer Ther.

[66] Fiorillo M, Tóth F, Brindisi M, Sotgia F, Lisanti MP (2020). Deferiprone (DFP) targets cancer stem cell (CSC) propagation by inhibiting mitochondrial metabolism and inducing ROS production. Cells.

[67] Delavar Mendi F, Sh. Saljooghi A, Ramezani M, Kruszynski R, Poupon M, Kucerakova M, Huch V, Socha P, Babaei M, Alibolandi M (2021). Five new complexes with deferiprone and N, N-donor ligands: evaluation of cytotoxicity against breast cancer MCF-7 cell line and HSA-binding determination. J Biomol Struct Dyn.

[68] Osman S, Raza A, Al-Zaidan L, Inchakalody VP, Merhi M, Prabhu KS, Abdelaziz N, Hydrose S, Uddin S, Dermime S (2021). Anti-cancer effects of Tranilast: an update. Biomed Pharmacother.

[69] Wu X, Liu C, Li Z, Gai C, Ding D, Chen W, Hao F, Li W (2020). Regulation of GSK3$$\beta$$/NRF2 signaling pathway modulated erastin-induced ferroptosis in breast cancer. Mol Cell Biochem.

[70] Khurana N, Chandra PK, Kim H, Abdel-Mageed AB, Mondal D, Sikka SC (2020). Bardoxolone-methyl (CDDO-me) suppresses androgen receptor and its splice-variant AR-V7 and enhances efficacy of enzalutamide in prostate cancer cells. Antioxidants.

[71] Nandi U, Onyesom I, Douroumis D (2021). An in vitro evaluation of antitumor activity of sirolimus-encapsulated liposomes in breast cancer cells. J Pharm Pharmacol.

[72] Jaffar NFN, Sakri MSM, Jaafar H, Rahman WFWA, Tengku TADA-A (2020). Evaluation of NMU-induced breast cancer treated with sirolimus and sunitinib on breast cancer growth. Asian Pac J Cancer Prev: APJCP.

[73] Ayoub NM, Ibrahim DR, Alkhalifa AE, Al-Husein BA (2021). Crizotinib induced antitumor activity and synergized with chemotherapy and hormonal drugs in breast cancer cells via downregulating met and estrogen receptor levels. Investig New Drugs.

[74] Li M, Li Y, Li S, Jia L, Du C, Li M, Li S, Galons H, Guo N, Yu P (2021). Co-delivery of F7 and crizotinib by thermosensitive liposome for breast cancer treatment. J Liposome Res.

[75] Scioli MG, Storti G, D’Amico F, Gentile P, Fabbri G, Cervelli V, Orlandi A (2019). The role of breast cancer stem cells as a prognostic marker and a target to improve the efficacy of breast cancer therapy. Cancers.

[76] Raikwar S, Jain SK (2020). Opportunities and challenges in breast cancer. Int J Pharm Life Sci.

[77] Aviles NMN, Mayol N, Gonzalez MT, Diaz A, Salgado IK, Silva W, Maldonado H (2019). Anticancer and chemosensitizing effects of WEE-1 kinase inhibitor MK-1775 in triple negative (MB-231) breast cancer cells. FASEB J.

[78] Lee A, Fraser SP, Djamgoz MB (2019). Propranolol inhibits neonatal NAV1. 5 activity and invasiveness of MDA-MB-231 breast cancer cells: effects of combination with ranolazine. J Cell Physiol.

[79] Kaur J, Kaur B, Singh P (2018). Rational modification of semaxanib and sunitinib for developing a tumor growth inhibitor targeting ATP binding site of tyrosine kinase. Bioorg Med Chem Lett.

[80] Diéras V, Bonnefoi H, Alba E, Awada A, Coudert B, Pivot X, Gligorov J, Jager A, Zambelli S, Lindeman GJ (2019). Iniparib administered weekly or twice-weekly in combination with gemcitabine/carboplatin in patients with metastatic triple-negative breast cancer: a phase ii randomized open-label study with pharmacokinetics. Breast Cancer Res Treat.

[81] Yasa INWT. The role of goserelin acetate in the management of pre-menopausal breast cancer. School of Surgical Oncology for General Surgeons XV; 2018. p. 49.

[82] Rupp T, Pelouin O, Genest L, Legrand C, Froget G, Castagné V (2021). Therapeutic potential of Fingolimod in triple negative breast cancer preclinical models. Transl Oncol.

[83] McLaughlin RP, He J, Van Der Noord VE, Redel J, Foekens JA, Martens JW, Smid M, Zhang Y, Van de Water B (2019). A kinase inhibitor screen identifies a dual CDC7/CDK9 inhibitor to sensitise triple-negative breast cancer to EGFR-targeted therapy. Breast Cancer Res.

[84] Abdulaziz NT, Mustafa YF (2021). Anticancer properties of hymecromone-derived compounds: a review. Int J Pharm Res.

[85] Gao H-L, Gupta P, Cui Q, Ashar YV, Wu Z-X, Zeng L, Lei Z-N, Teng Q-X, Ashby CR, Guan Y (2020). Sapitinib reverses anticancer drug resistance in colon cancer cells overexpressing the ABCB1 transporter. Front Oncol.

[86] Liu H, Dilger JP, Lin J (2021). Lidocaine suppresses viability and migration of human breast cancer cells: TRPM7 as a target for some breast cancer cell lines. Cancers.

[87] Chamaraux-Tran T-N, Mathelin C, Aprahamian M, Joshi GP, Tomasetto C, Diemunsch P, Akladios C (2018). Antitumor effects of lidocaine on human breast cancer cells: an in vitro and in vivo experimental trial. Anticancer Res.

[88] Chew NJ, Nguyen EV, Su S-P, Novy K, Chan HC, Nguyen LK, Luu J, Simpson KJ, Lee RS, Daly RJ (2020). FGFR3 signaling and function in triple negative breast cancer. Cell Commun Signal.

[89] Pennock ND, Martinson HA, Guo Q, Betts CB, Jindal S, Tsujikawa T, Coussens LM, Borges VF, Schedin P (2018). Ibuprofen supports macrophage differentiation, T cell recruitment, and tumor suppression in a model of postpartum breast cancer. J Immunother Cancer.

[90] Xia W, Zhang S, Li Y, Fan J, Liu B, Wang L, Peng X (2021). Ibuprofen-derived fluorescence inhibitor of COX-2 for breast cancer imaging, prevention and treatment. Dyes Pigments.

[91] Schobert R, Biersack B (2019). Chemical and biological aspects of garcinol and isogarcinol: recent developments. Chem Biodivers.

[92] Aggarwal V, Tuli HS, Kaur J, Aggarwal D, Parashar G, Chaturvedi Parashar N, Kulkarni S, Kaur G, Sak K, Kumar M (2020). Garcinol exhibits anti-neoplastic effects by targeting diverse oncogenic factors in tumor cells. Biomedicines.

[93] Balbuena-Rebolledo I, Padilla-Martínez II, Rosales-Hernández MC, Bello M (2021). Repurposing FDA drug compounds against breast cancer by targeting EGFR/HER2. Pharmaceuticals.

[94] Chen L, Long C, Nguyen J, Kumar D, Lee J (2018). Discovering alkylamide derivatives of bexarotene as new therapeutic agents against triple-negative breast cancer. Bioorg Med Chem Lett.

[95] Olden K, Breton P, Grzegorzewski K, Yasuda Y, Gause BL, Oredipe OA, Newton SA, White SL (1991). The potential importance of swainsonine in therapy for cancers and immunology. Pharmacol Ther.

[96] Li J-X, Bi Y-P, Wang J, Yang X, Tian Y-F, Sun Z-F (2018). JTC-801 inhibits the proliferation and metastasis of ovarian cancer cell SKOV3 through inhibition of the PI3K-AKT signaling pathway. Die Pharmazie-Int J Pharm Sci.

[97] Morimoto K, Kinoshita H (1998). Once-daily use of ofloxacin for prophylaxis in breast cancer surgery. Chemotherapy.

[98] Turner NC, Ro J, André F, Loi S, Verma S, Iwata H, Harbeck N, Loibl S, Huang Bartlett C, Zhang K (2015). Palbociclib in hormone-receptor-positive advanced breast cancer. N Engl J Med.

[99] Cauley JA, Norton L, Lippman ME, Eckert S, Krueger KA, Purdie DW, Farrerons J, Karasik A, Mellstrom D, Ng KW (2001). Continued breast cancer risk reduction in postmenopausal women treated with raloxifene: 4-year results from the MORE trial. Breast Cancer Res Treat.

[100] Thabet NM, Moustafa EM (2017). Synergistic effect of Ebselen and gamma radiation on breast cancer cells. Int J Radiat Biol.

[101] Tian J, Al Raffa F, Dai M, Moamer A, Khadang B, Hachim IY, Bakdounes K, Ali S, Jean-Claude B, Lebrun J-J (2018). Dasatinib sensitises triple negative breast cancer cells to chemotherapy by targeting breast cancer stem cells. Br J Cancer.

[102] Saeki T, Nomizu T, Toi M, Ito Y, Noguchi S, Kobayashi T, Asaga T, Minami H, Yamamoto N, Aogi K (2007). Dofequidar fumarate (MS-209) in combination with cyclophosphamide, doxorubicin, and fluorouracil for patients with advanced or recurrent breast cancer. J Clin Oncol.

[103] Swami S, Krishnan AV, Wang JY, Jensen K, Peng L, Albertelli MA, Feldman D (2011). Inhibitory effects of calcitriol on the growth of MCF-7 breast cancer xenografts in nude mice: selective modulation of aromatase expression in vivo. Hormones Cancer.

[104] Fry DW, Kraker AJ, McMichael A, Ambroso LA, Nelson JM, Leopold WR, Connors RW, Bridges AJ (1994). A specific inhibitor of the epidermal growth factor receptor tyrosine kinase. Science.

[105] Meijer M, Thygesen LC, Green A, Emneus M, Brasso K, Iversen P, Pukkala E, Bolin K, Stavem K, Ersbøll AK (2018). Finasteride treatment and male breast cancer: a register-based cohort study in four Nordic countries. Cancer Med.

[106] Elia SG, Al-Karmalawy AA, Nasr MY, Elshal MF (2021). Loperamide potentiates doxorubicin sensitivity in triple-negative breast cancer cells by targeting MDR1 and JNK and suppressing MTOR and Bcl-2: in vitro and molecular docking study. J Biochem Mol Toxicol.

[107] Wihandono A, Azhar Y, Abdurahman M, Hidayat S (2021). The role of lisinopril and bisoprolol to prevent anthracycline induced cardiotoxicity in locally advanced breast cancer patients. Asian Pac J Cancer Prev.

[108] O’Shaughnessy J, Osborne C, Pippen JE, Yoffe M, Patt D, Rocha C, Koo IC, Sherman BM, Bradley C (2011). Iniparib plus chemotherapy in metastatic triple-negative breast cancer. N Engl J Med.

[109] Barbosa RS, Dantonio PM, Guimarães T, de Oliveira MB, Alves VLF, Sandes AF, Fernando RC, Colleoni GW (2019). Sequential combination of bortezomib and wee1 inhibitor, MK-1775, induced apoptosis in multiple myeloma cell lines. Biochem Biophys Res Commun.

[110] de Nonneville A, Finetti P, Birnbaum D, Mamessier E, Bertucci F (2021). Wee1 dependency and pejorative prognostic value in triple-negative breast cancer. Adv Sci.

[111] Baloni P, Dinalankara W, Earls JC, Knijnenburg TA, Geman D, Marchionni L, Price ND (2021). Identifying personalized metabolic signatures in breast cancer. Metabolites.

[112] Jaragh-Alhadad LA, Harisa GI, Alanazi FK (2022). Development of nimesulide analogs as a dual inhibitor targeting tubulin and HSP27 for treatment of female cancers. J Mol Struct.

[113] Jang DK, Lee YG, Chae YC, Lee JK, Paik WH, Lee SH, Kim Y-T, Ryu JK (2020). GDC-0980 (apitolisib) treatment with gemcitabine and/or cisplatin synergistically reduces cholangiocarcinoma cell growth by suppressing the PI3K/Akt/mTOR pathway. Biochem Biophys Res Commun.

[114] Thabethe K, Adefolaju G, Hosie M (2013). An in vitro study of the effects of emtricitabine, tenofovir disoproxil fumarate and efavirenz on a breast cancer cell line, MCF-7. J Basic Appl Sci Res.

[115] Hobbs E, Lindquist E, Sullivan B, Yoon E, Yuan Y, Marx A, Willey J, Sun H, Layman R, Stover D, et al. Abstract OT-28-04: Neratinib and tepotinib combination in advanced breast cancer and inflammatory breast cancer patients with abnormal HER2 and c-Met pathway activity as measured by the CELsignia signaling pathway activity test. AACR, 2021.

[116] Lu P, Gu Y, Li L, Wang F, Yang X, Yang Y (2019). Retracted article: belinostat suppresses cell proliferation by inactivating Wnt/$$beta$$-catenin pathway and promotes apoptosis through regulating PKC pathway in breast cancer. Artif Cells Nanomed Biotechnol.

[117] Zuo Y, Xu H, Chen Z, Xiong F, Zhang B, Chen K, Jiang H, Luo C, Zhang H (2020). 17-AAG synergizes with Belinostat to exhibit a negative effect on the proliferation and invasion of MDA-MB-231 breast cancer cells. Oncol Rep.

[118] Gwynne WD, Shakeel MS, Girgis-Gabardo A, Hassell JA (2021). The role of serotonin in breast cancer stem cells. Molecules.

[119] Buxant F, Kindt N, Laurent G, Noël J-C, Saussez S (2015). Antiproliferative effect of dexamethasone in the MCF-7 breast cancer cell line. Mol Med Rep.

[120] Yao Q, Li J, Chen R, Yao Y, Xue J, Chen W, Lu W, Zhou T (2019). Preclinical PK/PD model for the combinatorial use of dexamethasone and sulpiride in the treatment of breast cancer. Acta Pharmacol Sin.

[121] Kaya Cakır H, Eroglu O (2021). In vitro anti-proliferative effect of capecitabine (Xeloda) combined with mocetinostat (MGCD0103) in 4T1 breast cancer cell line by immunoblotting. Iran J Basic Med Sci.

